# EMQN best practice guidelines for genetic testing in hereditary breast and ovarian cancer

**DOI:** 10.1038/s41431-023-01507-5

**Published:** 2024-03-05

**Authors:** Trudi McDevitt, Miranda Durkie, Norbert Arnold, George J. Burghel, Samantha Butler, Kathleen B. M. Claes, Peter Logan, Rachel Robinson, Katie Sheils, Nicola Wolstenholme, Helen Hanson, Clare Turnbull, Stacey Hume

**Affiliations:** 1 Department of Clinical Genetics, Children’s Health Ireland at Crumlin, Dublin, Ireland; 2 Sheffield Diagnostic Genetics Service, North East and Yorkshire Genomic Laboratory Hub, Sheffield Children’s NHS Foundation Trust Western Bank, Sheffield, UK; 3 UKSH Campus Kiel, Gynecology and Obstetrics, Institut of Clinical Chemistry, Institut of Clinical Molecular Biology, Kiel, Germany; 4 Manchester University NHS Foundation Trust, North West Genomic Laboratory Hub, Manchester, UK; 5 Central and South Genomic Laboratory Hub, West Midlands Regional Genetics Laboratory, Birmingham Women’s and Children’s NHS Foundation Trust, Birmingham, UK; 6 Ghent University Hospital, Center for Medical Genetics, Gent, Belgium; 7 HSCNI / Belfast Trust Laboratories, Regional Molecular Diagnostics Service, Belfast, Northern Ireland; 8 Leeds Teaching Hospitals NHS Trust, Genetics Department, Leeds, UK; 9 EMQN, Manchester, UK; 10 St George’s University Hospitals NHS Foundation Trust, Clinical Genetics, London, UK; 11 Institute of Cancer Research, London, UK; 12 University of British Columbia, Pathology and Laboratory Medicine, Vancouver, British Columbia Canada

**Keywords:** Cancer genomics, Cancer genetics

## Abstract

Hereditary Breast and Ovarian Cancer (HBOC) is a genetic condition associated with increased risk of cancers. The past decade has brought about significant changes to hereditary breast and ovarian cancer (HBOC) diagnostic testing with new treatments, testing methods and strategies, and evolving information on genetic associations. These best practice guidelines have been produced to assist clinical laboratories in effectively addressing the complexities of HBOC testing, while taking into account advancements since the last guidelines were published in 2007. These guidelines summarise cancer risk data from recent studies for the most commonly tested high and moderate risk HBOC genes for laboratories to refer to as a guide. Furthermore, recommendations are provided for somatic and germline testing services with regards to clinical referral, laboratory analyses, variant interpretation, and reporting. The guidelines present recommendations where ‘**must**’ is assigned to advocate that the recommendation is essential; and ‘**should**’ is assigned to advocate that the recommendation is highly advised but may not be universally applicable. Recommendations are presented in the form of shaded italicised statements throughout the document, and in the form of a table in supplementary materials (Table S4). Finally, for the purposes of encouraging standardisation and aiding implementation of recommendations, example report wording covering the essential points to be included is provided for the most common HBOC referral and reporting scenarios. These guidelines are aimed primarily at genomic scientists working in diagnostic testing laboratories.

## 1.0 Introduction

Global estimates indicate that breast cancer (BC) is the most common female cancer diagnosis, accounting for approximately 24.8% of all female cancer diagnoses worldwide, and is the leading cause of female cancer death. Ovarian cancer (OC) accounts for approximately 4.4% of female cancer diagnoses. It is the eighth most common cancer occurring in women and the 2^nd^ most common cause of gynaecological cancer death worldwide [1].

The general population incidence of male BC is ~1/1000 and represents approximately 1% of all BC cases [2].

Multigene panel (MGP) studies of known HBOC genes in large mixed patient cohorts suggest a pathogenic and/or likely pathogenic variant (PV) prevalence of approximately 8% in BC cases and 16% in OC cases; with PVs in *BRCA1* and *BRCA2 (BRCA1/2)* accounting for approximately 50% and 80% of the total PVs respectively (Table S1) [3, 4].

Prevalence, associated risk and absolute risk data for PVs in genes most commonly present on HBOC MGPs are presented in Table S1 and Table S2. Where available, data has been obtained from large prospective studies, and from studies comprising large numbers of patients tested by clinical testing laboratories. Table S1 presents data for known HBOC genes for which there is definitive data in support of an associated cancer risk. The genes listed in Table S2 are those for which the current evidence is limited or conflicting. In Tables S1 and S2, data on associated cancers other than BC and OC are provided for *BRCA1*, *BRCA2* and *PALB2* only, as PVs in these high risk genes are the ones most frequently detected.

### 1.1 HBOC genes

See Supplementary Material Section S1.1 for background information on high risk, moderate risk and candidate HBOC genes.

### 1.2 Multiple susceptibility risk alleles and polygenic risk scores

It is well established that cancer risk is modified by family history (*BRCA1/2* [5]; *PALB2* [6]; *RAD51C* and *RAD51D (RAD51C/D)* [7]). Advances have been made towards improving the accuracy of cancer risk estimation through the use of polygenic risk scores (PRS). Guidelines state that further validation is required before diagnostic HBOC services routinely use PRS to inform clinical management [8], references therein); however, PRS testing is now available in some commercial laboratories, and the 313-SNP PRS score [9] has been incorporated into BOADICEA/CanRisk. Clinical trials to validate PRS models and use in clinical practice are ongoing [10–12].

### 1.3 Tumour pathology

BC is a very heterogeneous disease with many histological types and subtypes. An association of intrinsic tumour subtype of BC with PVs in each of 9 HBOC susceptibility genes *BRCA1, BRCA2, PALB2, ATM, BARD1, CHEK2, RAD51C, RAD51D, TP53* has recently been reported [13]. While significant heterogeneity in the distribution of intrinsic subtypes by gene was observed, as consistent with previous studies; taken together, PV in the genes studied were generally associated with triple negative (TN) disease and/or a higher tumour grade.

High grade serous epithelial ovarian cancer (HGSOC) comprises more than 70% of all OC [14]. *BRCA1* or *BRCA2* PVs have been reported in 15−22% of HGSOC [15–17], and more than 70% of these tumours are grade 3 carcinomas [18]. The mutational frequency for the non-serous cancer subtypes (ie mainly endometrioid and clear cell) is estimated to be lower than 10% and even less frequent in mucinous carcinomas [19].

There is evidence to suggest a Lynch-related subtype of OC comprising mostly endometrioid or clear cell histology [20].

#### 1.3.1 PARP inhibitors

Poly (ADP-ribose) polymerases (PARP) are important enzymes in DNA damage repair mechanisms. Tumour cells with homologous recombination deficiency (HRD) are sensitive to PARP inhibitors (PARPi) which attack tumours with defective homologous recombination repair (HRR) proteins by a concept termed ‘synthetic lethality’. Clinical trials have shown that PARPi are beneficial in the treatment of patients with HRD tumours including those with germline and somatic *BRCA* PVs. Initially, PARPi maintenance therapy was limited to HGSOC. More recently, clinical trials and licensing have included PARPi for the treatment of *BRCA1/2*-related BC, metastatic prostate cancer, pancreatic cancer, with others to follow (many reviews available for example [21]),.

Failure to respond to PARPi has been observed in more than 40% of OC patients and acquired resistance to PARPi is also common. Causative mechanisms are extensively reviewed [22, 23].

## 2.0 Methods

An update of the guidelines was deemed necessary due to the rapidly evolving and increasingly complex scientific nature of genetic testing since 2007 (https://www.emqn.org/wp-content/uploads/2017/07/EMQN_BRCAguidelines2007.pdf). To achieve a broad expert consensus, 9 expert laboratory representatives from 9 centres across the UK, EU and Canada were invited to share their expertise in genetic testing for HBOC. The representatives met at the inaugural teleconference on February 1st 2019 to discuss the drafting framework and thereafter conducted virtual meetings over regular intervals between March 2019 and September 2020 culminating in the first draft of the guidelines.

The following points were discussed during the virtual meetings, and group consensus was achieved:

- Referral criteria
- Testing strategy and technologies
- Genes tested and associated risks
- Somatic testing
- Variant interpretation
- Referral pathways
- Reporting standards

Thereafter, the three co-first authors optimised and consolidated the draft document through e-mail correspondence and teleconferences between October 2020 and June 2022, while taking into account ongoing developments published during this time. Clinical expertise was sought, and two clinical expert representatives were invited to review and contribute to the draft document at the start of July 2021. Thereafter, all eight representatives (6 laboratory and 2 clinical) were consulted by the three co-first authors on a regular basis for feedback and contributions as the draft document progressed.

The draft document was made available through EMQN to a community of 489 participating laboratories in the EMQN-organised external quality assessment (EQA) schemes for HBOC panel testing, HBOC targeted BRCA testing, Ovarian and prostate cancer (v Somatic) [PARPi] and Ovarian, Breast, prostate and pancreatic cancers (v Germline) [PARPi] for comments. The community consultation period was held between 8th June and 4th July 2022. During the same period, the EMQN Management Group reviewed the document and made suggestions for clarity and content improvements. Based on feedback collected and evaluated during the EMQN community consultation period, the three co-first authors resumed their efforts via e-mail correspondence and teleconferences to produce the final version of the guidelines. All 11 representatives reviewed and approved the final draft of the guidelines in February 2023 and the consensus recommendations for genetic testing for hereditary breast and ovarian cancer were finalised.

## 3.0 HBOC referral pathways

### 3.1 Referral for germline testing

Historically, clinical genetics was the main referral pathway for HBOC testing using risk assessment tools such as BOADICEA/CanRisk [24, 25], Manchester Scoring method [26] and modified Chompret criteria for Li-Fraumeni syndrome [27]. Most individuals were referred for testing due to a personal and family history of HBOC. More recently, international initiatives have been established to provide direct or “mainstream” access to HBOC testing, often based on high likelihood of a PV. Genetic testing through a non-genetics specialty as part of the cancer pathway (mainstreaming) is anticipated to increase the efficiency of access to genetic testing by streamlining patient pathways. Mainstreaming includes discussion of a genetic test with the patient, including an overview of potential implications for the individual and wider family. Then requesting the test, returning results and ensuring an onward referral to Clinical Genetics where a PV is identified, or where no PV is identified but there remains concern about the family history of cancer [28]. With appropriate training and education, test requests from specialists other than geneticists are encouraged to promote timely, appropriate testing.

Regardless of the route taken, it is important that appropriate genetic counselling is integral to all genetic testing pathways (targeted or full screen).

**Table:** 

1. *However, due to the specific discussions required in the case of predictive genetic testing in individuals unaffected with cancer, appropriate genetic counselling* ***must*** *be an integral part of the process for individuals undergoing predictive testing*.	

Although testing of affected individuals represents the majority of germline testing, testing may also be available to unaffected individuals with no available living affected relative, but with a family history of HBOC meeting appropriate eligibility criteria (e.g., ≥10% likelihood of heterozygosity for a PV ([29] in the UK)). As an alternative to testing an unaffected individual and subject to local or national policies, testing on stored tissue from a deceased relative may be offered. This option may provide a more definitive outcome compared to that offered by the screening of an unaffected relative. Such testing can also aid cosegregation analysis.

In rare cases, a HBOC PV may be detected outside of the established HBOC clinical testing pathways, in these cases:

**Table:** 

2. *Clinical genetics involvement* ***should*** *be recommended for the small number of patients who have secondary or incidental findings e.g. copy number loss or gain identified by array analysis, or PVs identified through whole genome/exome/direct-to-consumer tests*.	

### 3.2 Referral for somatic tumour testing

*BRCA1/2* tumour testing is often recommended for all women diagnosed with ovarian, fallopian tube and primary peritoneal cancer for the purpose of directing PARPi therapy [30]; [29], dependent on local policies and licensing arrangements.

**Table:** 

3. *In addition to ovarian tumour testing, breast tumour and other HBOC-related tumour testing* ***should*** *also be utilised where PARPi therapies are licensed and available*.	

## 4.0 Genetic testing

Clinical referrals are received for both treatment decisions and determination of risk of hereditary cancer predisposition. Prior to implementation of a HBOC genetic testing service:

**Table:** 

4. *Laboratories* ***must*** *ensure that the performance of analytical methods meet the required standard for diagnostic testing through initial and ongoing internal validation/verification (Medical Laboratory Accreditation–ISO 15189:2022* [31]*; and appropriate EQA participation*.	

### 4.1 Diagnostic testing

Historically, indirect screening techniques may have been used as economical gene scanning methods, for example: Denaturing High Performance Liquid Chromatography. Sanger sequencing of the coding and splice site regions for single nucleotide variants (SNVs), plus copy number variant (CNV) analysis was traditionally utilised for direct PV detection. Knowledge of the past test history of a referred patient is important, as depending on the analytical sensitivity of the test performed at the time, retesting with newer test methodologies may be warranted [32]. The decreasing cost of next generation sequencing (NGS), alongside improved knowledge of the risk contributions of other genes, has permitted the rapid adoption of HBOC MGP analysis in many clinical genetics laboratories.

The selection of genes to include on MGPs should be driven by clinical utility, with each centre deciding on whether to implement pan-cancer gene panels or smaller phenotype-specific panels. Large MGPs may simplify workflows; however, laboratories and clinicians should be aware that as the number of genes on a panel increases, there is an increased risk of discovering variants not affiliated with the presenting disease. This can be reduced by bioinformatically evaluating only those genes associated with the cancer type.

Several resources are available to aid establishment of gene testing panels, notably: the Clinical Genome Resource (ClinGen) [33], Clinical Domain Working Groups formed by the Clinical Genome Resource [33–36]; PanelApp, a Genomics England crowd sourcing tool to facilitate the sharing and evaluation of gene panels in the scientific community (https://panelapp.genomicsengland.co.uk/) as well as recommendations from the National Comprehensive Cancer Network [8].

Testing of high risk HBOC genes (Table S1) is recommended.

**Table:** 

5. *As a minimum, laboratories* ***must*** *ensure that a HBOC diagnostic testing service (internal or via an external testing laboratory referral) includes analysis of high risk genes: BRCA1/2 and PALB2*.	

According to national guidelines, each jurisdiction should establish whether their panels will include only high risk genes or also offer moderate risk genes. Consideration should be given to regional recommendations for surveillance, surgical and pharmacological options for patients with a PV in a moderate or high risk gene (there are currently no surveillance recommendations for PVs in low-risk genes or those with sparse/conflicting evidence). Various guidelines including [8] (USA) [29]; (UK); GC-HBOC (https://www.health-atlas.de/projects/2) (Germany); eviQ (https://www.eviq.org.au/) (Australia) may be used as a reference point for clinical actionability. As actionable genes are changing frequently:

**Table:** 

6. *Laboratories* ***must*** *remain vigilant with current scientific literature and guideline updates to ensure MGPs remain current*. 7. *Laboratories* ***should*** *ensure that a HBOC diagnostic testing service (internal or external testing laboratory referral) provides analysis of intronic regions known to contain recurrent PVs (e.g., BRCA1 c.213-11T*>*G* [37]*)*. 8. *Reportable variants detected in genes with associated pseudogenes (for example, CHEK2 and PMS2)* ***must*** *be checked for specificity prior to reporting*.	

Historically, detection of CNVs relied solely on techniques such as multiplex ligation-dependent probe amplification (MLPA), quantitative real-time PCR or long-range PCR. The use of validated normalised NGS read depth is now commonly used for CNV analysis [38, 39]. Knowledge of the gene-specific contribution of CNVs helps inform testing strategy, which should aim to maximise clinical sensitivity: Laboratories may refer to [40] for recent CNV prevalence data.

**Table:** 

9. *Laboratories* ***should*** *have policies in place for determining which genes are analysed for CNVs*. 10. *As a minimum, laboratories* ***must*** *ensure that a HBOC diagnostic testing service (internal or external testing laboratory referral) provides CNV analysis for BRCA1/2*.	

#### 4.1.1 FFPE tissue testing

Formalin-fixed paraffin embedded (FFPE) testing may be offered for two clinical scenarios: HGSOC/other eligible tumour types for PARPi treatment eligibility, and deceased index patient testing in high risk families where no affected relative is available for testing. It is important to note however, that detection of germline and somatic CNVs and larger indels in FFPE tissue is challenging, as many NGS pipelines are not yet capable of achieving adequate sensitivity.

##### 4.1.1.1 HGSOC/other eligible tumour types for PARPi treatment eligibility (tumour tissue)

Analysis of ovarian tumour samples allows the detection of a *BRCA1/2* PV in approximately 15% of patients, including ~7% with a somatic-only variant [41]. Somatic variants are frequently present at lower levels; therefore, NGS analysis requires a higher sequence depth to achieve acceptable analytical sensitivities. Acceptable read depths will vary with each technology and between different panel libraries.

**Table:** 

11. *Laboratories* ***must*** *establish the assay limit of detection (LoD) and analytical sensitivity for all categories of variant types through appropriate validation*. 12. *The percentage neoplastic cell content assessment* ***must*** *be integral to tumour tissue analysis*. 13. *The neoplastic cell content percentage* ***should*** *be at least twice the validated LoD of the assay*.	

For tumour testing, the general technical challenges of FFPE tissue NGS analysis are further compounded by low variant allele frequency (VAF) due to variable percentage neoplastic content and tumour heterogeneity [37]. As a result, both somatic and germline CNVs/larger indels (<50 bp) may be missed [42, 43]. Furthermore, a recent study has reported 3.1% of true germline pathogenic variants to be absent from filtered tumour-detected variants [44].

In order to ensure that optimal analytical sensitivities are achieved:

**Table:** 

14. *Paired germline and tumour analysis* ***should*** *ideally be performed, with germline testing involving full gene panel analysis as appropriate for the tumour type, in addition to analysis of larger indels and CNVs*. 15. *If the laboratory protocol is for tiered testing beginning with somatic tissue; as a minimum, targeted germline follow-up testing* ***should*** *be performed to confirm any detected somatic PVs with VAF* > *30% (SNVs) or* > *20% (small indels)* [44]*, and to detect larger indels and CNVs which may have a lower LoD*.	

In addition to the analysis of *BRCA1/2* in tumour samples, some assays also include measurement of HRD by NGS analysis of genome-wide SNPs, including assessment of loss of heterozygosity (LOH) and/or other markers of genomic instability. Various HRD assays are available (reviewed in [45]). Efforts are underway to standardise HRD testing for clinical application based on demonstration of response to PARPi in clinical trials (e.g., Friends of Cancer Research: https://friendsofcancerresearch.org/hrd/). Thus, tumour samples exhibiting HRD detected on an assay for which response to PARPi has been demonstrated in clinical trials, or on one that is benchmarked to such an assay (HRD positive); for example, due to *BRCA1* promoter hypermethylation or a PV in other HRR genes, are eligible for PARPi even if no PV is detected in *BRCA1/2*.

##### 4.1.1.2 Deceased index patient germline testing

Non-neoplastic material is preferred for this analysis but if not available, predominantly non-neoplastic material with low level neoplastic cell content (below the LoD of the assay) is acceptable. Adequate test sensitivity may be achieved with lower NGS coverage depth in the case of deceased index patient testing on non-neoplastic tissue; however, failure rates due to technical challenges associated with FFPE tissue analysis may be significant [46].

### 4.2 Specific Variant Testing (predictive testing)

Where a causative PV has been identified in an index case, predictive testing for at-risk family members can be offered. It is important to note that if the index patient testing occurred many years ago, the patient report may refer to historical nomenclature i.e based on numbering starting in 5’UTR or use of IVS±n for intronic variants.

**Table:** 

16. *Confirmation of the correct gene region to be targeted* ***must*** *be integral to the predictive testing process*. 17. *A familial positive control where available (ideally first degree relative)* ***should*** *be included with each assay to minimise a false negative result*. 18. *Primer/probe sequences* ***must*** *be checked regularly against recent large population studies (e.g. gnomAD) to ensure there are no reported SNVs which could potentially cause non-amplification of one allele*. 19. *To minimise the risk of incidental findings, analysis for a specific variant* ***should*** *be limited to a defined region of the gene containing the variant, including when NGS technology is used*.	

## 5.0 Variant interpretation

### 5.1 Framework

The IARC Unclassified Genetic Variants Working Group published guidelines for interpreting and reporting germline variants in cancer predisposition genes [47]. They proposed a standardised five-tier classification system based on the likelihood of pathogenicity and this system has been widely adopted e.g., InSiGHT (https://www.insight-group.org/), ENIGMA (https://enigmaconsortium.org/).

In 2015, the American College of Medical Genetics and Genomics (ACMG) and Association for Molecular Pathology (AMP) published a five-tier variant classification system applicable to variants in all Mendelian genes, which has been implemented internationally [48].

The UK Association for Clinical Genomic Sciences (ACGS) routinely publishes updated best practice guidelines for variant interpretation, providing additional information to assist with the application of the ACMG/AMP guidelines [49]. The UK Cancer Variant Interpretation Group (CanVIG) has published a detailed specification for variant interpretation in cancer susceptibility genes using the ACMG/AMP and ACGS framework [50]. Current versions and multiple other useful resources are available on their website https://www.cangene-canvaruk.org/canvig-uk.

ClinGen in collaboration with others such as ENIGMA for *BRCA1/2*, have set up gene, disease and evidence-specific expert groups to enhance the variant classification framework (Variant Curation Expert Panels (VCEP)). Gene-specific guidelines have been published for *PTEN*, *CDH1*, *TP53, MMR, ATM* with many more in development (e.g., *BRCA1* and *BRCA2*). Further refinement of ACMG/AMP classification guidelines have been published for several evidence sources, for example, protein truncating variants (PTVs), functional studies, and de novo variants (https://clinicalgenome.org/working-groups/sequence-variant-interpretation/#heading_documents).

Guidelines based on the original ACMG/AMP guidelines [48] have recently been established for the interpretation of SNVs in non-coding regions [51].

**Table:** 

20. *Germline variant classification* ***must*** *be performed according to ACMG/AMP guidelines* [48] *(or national/local approved guidelines) with the use of gene-specific and expert guidelines where available e.g. ClinGen VCEP, CanVIG or ENIGMA*.	

Separate guidelines for somatic variant interpretation have been established [52]; however:

**Table:** 

21. *Variants identified during somatic testing* ***should*** *be classified using ACMG/AMP germline guidance as well as somatic tiering, as certain variants (in particular BRCA1/2) have a high likelihood of germline origin* [17, 53]. 22. *Variants identified* ***should*** *be submitted to a database such as ClinVar* [54] *to aid subsequent review and classification*.	

### 5.2 Considerations for variant review and reclassification

Ideally variant classifications, particularly variant of uncertain significance (VUS) should be regularly reviewed; however, this will depend on local guidance and resources, and laboratories should have appropriate policies and procedures in place. Laboratories may refer to https://enigmaconsortium.org.

**Table:** 

23. *Laboratories* ***must*** *consider a variant review after a laboratory-defined period of variant classification has lapsed (e.g*. > *12 months) and:* i. *Re-identification of the variant in the laboratory OR* ii. *Following the release of new information from another laboratory or from the scientific literature/public database (ClinVar* [54]*/Decipher (*https://www.deciphergenomics.org*) OR* iii. *Following new phenotypic information on the proband or family member OR* iv. *Following a request from the referring consultant e.g. prior to cascade testing, risk-reducing surgery etc*. 24. *Where reclassification is likely to change clinical management i.e. from VUS to likely pathogenic (LP) or LP to VUS, and particularly if the evidence is not publicly available, notification* ***should*** *be provided to relevant other diagnostic laboratories and appropriate healthcare professionals to ensure consistent patient management* [55].	

### 5.3 HBOC specific variant interpretation

It is important to consider naturally occurring alternative splicing for analysed genes. Invariant splice or nonsense variants leading to naturally occurring in-frame RNA isoforms may rescue protein function; therefore these should be considered VUS unless proven otherwise e.g. PTVs in *BRCA1* exons 9 or 10 (MANE select numbering), or in *BRCA2* exon 12 (https://enigmaconsortium.org). Splice variants affecting NAGNAG (tandem acceptor) sites are predicted to produce an in-frame transcript. Predicted nonsense or frameshift variants downstream of codon 1854 (BRCA1 protein) [56] and codon 3309 (BRCA2 protein) [56, 57] are highly unlikely to be clinically important https://enigmaconsortium.org. PTVs in the first and last exons and 50 bp of the penultimate exon are usually not subject to nonsense mediated decay. Frameshift variants in the last exon may cause a protein extension and can be subject to non-stop mediated decay.

The *BRCA1* c.5096G>A p.(Arg1699Gln) missense variant has been shown to have an intermediate risk for BC and OC compared to other PVs and specific clinical management guidelines have been published for this variant [58]. Other putative reduced penetrance variants may be identified from Fanconi anaemia (FA)-like cases or postulated from functional assays. However, large case-control studies (e.g., [59]) are required to confirm any significant reduction in penetrance; and in the absence of such studies, individuals should be managed appropriately based on their personal and family history.

For *TP53* variants:

**Table:** 

25. *The presence of Clonal Haematopoiesis of Indeterminate Potential (CHIP)/mosaicism* ***must*** *be considered for all cases for which (i) there is no familial transmission evident AND (ii) VAF* < *40% AND (iii) the phenotype is not supportive of LFS* [60]. 26. *Laboratories* ***should*** *perform analysis to exclude/confirm CHIP*.	

Testing of germline tissues e.g. normal tissue from tumour block, cultured fibroblasts from skin biopsy or hair follicle is recommended. The presence of germline mosaicism should be considered [61].

Pending definitive evidence on the contribution of missense variants to HBOC:

**Table:** 

27. *Caution* ***should*** *be exercised when reporting missense variants in PALB2, RAD51C, RAD51D, CHEK2 and ATM genes, where disease has been predominantly associated with PTVs to date*.	

## 6.0 Reporting

Laboratories must ensure that interpretation and reporting meet the required standard for diagnostic testing (Medical Laboratory Accreditation-ISO 15189:2022 or equivalent); and that these elements are evaluated through regular participation in EQA where this is available.

### 6.1 General report features

See [62, 63].

See comprehensive guidelines on the reporting of variants, including class 3 (VUS) and example reporting formats [49].

**Table:** 

28. *Guidelines used for variant interpretation* ***must*** *be clearly referenced in clinical reports*. 29. *Furthermore, variant classification evidence* ***must*** *be available to service users, preferably as part of the report, or minimally upon request*.	

### 6.2 Clinical recommendations

It is recognised that jurisdictions may have differing policies for describing clinical options in genetic reports. Information in relation to options for further testing, surveillance, risk-reducing surgery, targeted drug therapy may be included in reports, or communicated during a clinical consultation.

**Table:** 

30. *As a minimum, reports* ***must*** *recommend referral of patients for appropriate clinical management and genetic counselling in cases where a germline PV/reportable VUS has been identified*. 31. *Where an elevated residual risk remains after testing, and depending on local reporting policy: Reports* ***should*** *advise that clinical management should be dependent on personal and family history*.	

### 6.3 Technical report features

See [64, 65].

**Table:** 

32. *Technical information including NGS/MLPA kit details and version number, sequencing chemistry, bioinformatics pipeline, LoD and analytical sensitivity* ***must*** *be available via the report*.	

For the classification and reporting of variants, the authors recommend use of MANE Select and MANE Plus Clinical transcripts (ideally based on GRCh38) for standardisation [66].

**Table:** 

33. *Each variant reported* ***must*** *be described using HGVS nomenclature, including the clinically appropriate transcript (e.g. MANE select and/or MANE Plus Clinical) and version number, zygosity (germline variants), and include details of the predicted effect on the protein where appropriate*.	

#### 6.3.1 Diagnostic testing

The gene regions and types of variants covered by the test must be stated in the report. If some types of variants (for example, CNVs) or gene regions (for example, introns with common PVs) have not been adequately assessed, this must be clearly indicated. Analytical sensitivity should be as high as possible, and indicated in the report [31].

**Table:** 

34. *The report* ***must*** *state the test scope and assay limitations, and refer the patient elsewhere for further testing/analysis of additional genes implicated in HBOC as appropriate, should the analytical and/or clinical sensitivity fail to reach the required laboratory-determined threshold/standard*. 35. *For TP53 variants fitting the criteria for consideration of CHIP/mosaicism (see Section* 5.3*), reports* ***must*** *clearly state the risk of CHIP if further work is not performed*, *or CHIP has been experimentally excluded/confirmed*.	

As appropriate depending on local practice and policies, reports may recommend that the patient be referred for clinical surveillance/management in addition to recommending appropriate genetic counselling.

To aid VUS reporting decisions, laboratories may find it helpful to refer to the gradient of different VUS categories obtained using the ACMG/AMP scoring criteria and a Bayesian approach [49].

**Table:** 

36. *Variant classes not reported* ***must*** *be clearly stated on the report*.	

Example wording: *Benign/likely benign variants and some VUS with limited or conflicting information are not reported*.

**Table:** 

37. *If a PV is identified in a patient where consanguinity has been noted, the report* ***should*** *mention the risk of autosomal recessive (AR) disease e.g., FA and offer to test any consanguineous partner*.	

#### 6.3.2 Cascade testing

**Table:** 

38. *The extent of the gene region analysed* ***must*** *be clear in the report. For example, a statement that targeted variant analysis has been performed, and/or use of HGVS nomenclature to describe the genotype, and/or genomic coordinates and genome build (ideally GRCh38)*. 39. *PV-absent reports* ***must*** *state the test limitations if DNA from a familial positive control is not available for inclusion in the analysis* (see Section 4.2)	

### 6.4 Specific scenario report interpretation recommendations (Recommendation 40)

Example report wording covering the essential recommended points to be included is shown in italics within shaded areas below. Abbreviations are present for the purpose of space constraint only and are not intended to be incorporated into clinical reports.

#### 6.4.1 Diagnostic test for an eligible individual diagnosed with an HBOC-related cancer

6.4.1.1a Testing outcome: PV detected - high risk gene & variant

**Table:** 

*This result is consistent with (likely pathogenic) or confirms (pathogenic) a genetic* *diagnosis of GENE-related cancer susceptibility. The patient has a* *high risk of developing further GENE-related cancers (females) (or* *males: the patient is at risk of developing further GENE-related* *cancers) and should be managed appropriately*. *Each of their offspring would be at 50% risk of inheriting this* *variant and genetic predisposition to GENE-related cancers. Other* *relatives are also at increased risk of this disorder. We recommend* *referral for appropriate clinical management and genetic counselling* *where predictive and diagnostic testing for this variant in their* *relatives can be arranged as appropriate*.	

*6.4.1.1b Testing outcome: PV detected - reduced penetrance variant or moderate risk gene*

**Table:** 

*This result is consistent with (likely pathogenic) or confirms (pathogenic) a genetic diagnosis* *of GENE-related cancer susceptibility. This variant or gene is* *associated with reduced or moderate penetrance <add reference >*. *The patient is at risk of developing further GENE-related cancers and* *should be managed appropriately based on their personal and family* *history*. See 6.4.1.1a for further wording on risk to family members and associated recommendations.	

*6.4.1.2 Testing outcome: PV not detected*

**Table:** 

*No PV has been detected in these genes. This result reduces the* *chance of, but does not completely exclude a diagnosis of HBOC*.	

*6.4.1.3 Testing outcome: VUS detected*

**Table:** 

*This finding in isolation is insufficient to justify a change in clinical* *management. To aid variant reclassification, further evidence is* *required. We recommend referral for appropriate clinical management* *and genetic counselling (for familial segregation analysis/RNA studies/* *etc, if appropriate). Predictive testing is not indicated for relatives*. *Further evidence may become available for this variant in the future: if* *new clinical decisions based on this variant are required for this family*, *please contact the laboratory to request a review of this variant*. Note: Where a VUS is detected but not reported, see 6.4.1.2 (Testing outcome: PV not detected)	

#### 6.4.2 Test for an individual without an HBOC-related cancer, who is eligible due to their family history

Testing of eligible individuals without a HBOC-related cancer may be performed in cases where there is no living affected relative available, or no deceased affected relative with tissue material available for testing.

*6.4.2.1 Testing outcome: PV detected*

**Table:** 

*This individual has a high risk (if high risk gene, or “increased risk” if* *male or moderate risk gene or reduced penetrance variant) of* *developing GENE-related cancers and should be managed appropriately*. See 6.4.1.1a/b for further wording. For males see also 6.4.5.2.2.	

*6.4.2.2 Testing outcome: PV not detected*

**Table:** 

*This result reduces the chance of, but does not completely exclude* *the possibility that this individual will develop autosomal dominant (AD) HBOC. We cannot* *exclude the possibility that a PV in the tested genes or a PV in* *another cancer susceptibility gene is segregating in this family. We* *recommend testing a sample from an affected relative, if available*.	

*6.4.2.3 Testing outcome: VUS detected*

**Table:** 

*This finding in isolation is insufficient to justify change in clinical* *management. Based on family history and uncertainty* *surrounding this variant, this result is inconclusive and this individual’s* *cancer risk should be determined based on their personal and family* *history. We cannot exclude the possibility that an alternate PV in the* *tested genes or a PV in another cancer susceptibility gene is* *segregating in this family. We recommend testing a sample from an* *affected relative, if available. Predictive testing is not indicated for this* *variant*. See also 6.4.1.3. for further wording.	

#### 6.4.3 Rare testing outcomes from scenarios 6.4.1 and 6.4.2

*6.4.3.1 Transheterozygosity for BRCA1&2 PV*

Such cases should be reported in the same way as for *BRCA1/2* heterozygotes. See 6.4.1.1a/b/6.4.2.1. for further wording.

**Table:** 

*Additional example wording: We recommend familial segregation* *analysis to determine the origin of each variant, as both maternal* *and paternal relatives may be at risk of HBOC and/or related cancers*.	

*6.4.3.2 Biallelic BRCA1/BRCA2/PALB2 PV*

Based on reported FA cases to date, at least one of the alleles is likely to have reduced penetrance with partial function. Referral for appropriate clinical management and genetic counselling is an essential prerequisite for all cascade testing, due the complexities of determining the cancer risk associated with a potentially reduced penetrance allele. Reports should recommend familial segregation analysis to determine the origin of each variant, as both maternal and paternal relatives may be at risk of HBOC and/or related cancers.

#### 6.4.4 BRCA1/2 somatic/germline analysis for PARPi treatment in HGSOC/other eligible tumour types

##### *6.4.4.1 Reporting recommendations*

See tumour testing reporting guidelines [52, 67–69].

**Table:** 

41. *Referral for appropriate clinical management and genetic counselling* ***must*** *be recommended for all reportable variants identified during germline testing*. 42. *It* ***must*** *be clear from the report whether the result is from the analysis of tumour and/or germline tissue*. 43. *As a minimum, somatic test reports* ***must*** *recommend germline testing of the detected reportable variant if VAF is in the laboratory established germline range eg.,* > *30% (SNVs)*, > *20% (small indels)* [44]. 44. *The percentage neoplastic content* ***must*** *be stated on somatic test reports, and flagged if below the laboratory-determined acceptable threshold; as this is critical information for when a PV is not detected*. 45. *The report* ***must*** *clearly state whether analysis for CNVs/larger indels has been performed*. 46. *If CNV/larger indel analysis has not been completed on the tumour sample, then germline CNV/larger indel testing for BRCA1/2* ***should*** *be recommended (unless HRD is not detected on an assay for which response to PARPi has been demonstrated in clinical trials, or on one that is benchmarked to such an assay [HRD negative]), and the limitations of somatic-only testing clearly stated*. 47. *If germline testing has not identified a reportable variant, then somatic testing* ***should*** *be recommended, and the limitations of germline-only testing clearly stated*. 48. *As applicable, reports* ***should*** *refer to PARPi therapy rather than brand names*	

If *BRCA1/2* testing has not identified a reportable variant, then HRD testing may be recommended if available, to identify tumours eligible for PARPi.

*6.4.4.2 HGSOC/other eligible tumour types testing for PARPi Outcomes*

*6.4.4.2.1 Somatic/germline BRCA1/2 PV detected (and HRD positive, if done)*

**Table:** 

*This patient has an increased likelihood of benefitting from PARPi* *therapy*.	

If somatic only testing has been completed, germline analysis should be performed. If PV is present in the germline - see 6.4.1.1a/b

*6.4.4.2.2 Somatic/germline BRCA1/2 PV not detected (and HRD negative, if done)*

**Table:** 

*Based on this result in isolation and current licensing, PARPi* *therapy is not indicated*.	

Where germline only or somatic only testing has been performed, see 6.4.4.1 for further testing recommendations.

*6.4.4.2.3 Somatic/germline BRCA1/2 PV not detected and HRD Positive*

**Table:** 

*This patient has an increased likelihood of benefitting from PARPi* *therapy*.	

*6.4.4.2.4 Somatic/germline BRCA1/2 VUS detected and HRD negative*

**Table:** 

*Based on the result and current licensing, PARPi therapy is not* *currently indicated*. See also 6.4.4.1 for further testing recommendations.	

*6.4.4.2.5 Somatic/germline BRCA1/2 VUS detected and HRD positive*

**Table:** 

*This patient has an increased likelihood of benefitting from PARPi* *therapy*. If the variant is present in the germline - see 6.4.1.3.	

#### 6.4.5 Cascade screening for a PV

##### *6.4.5.1 Confirmatory testing*

For example, testing for a familial GENE PV in a patient with an HBOC-related cancer (high prior risk).

*6.4.5.1.1 GENE PV detected*

**Table:** 

See example wording 6.4.1.1a/b.	

*6.4.5.1.2 GENE PV not detected*

It is recommended that where appropriate, such cases are followed up according to laboratory policies and procedures in place for unexpected findings.

**Table:** 

*This result indicates that the familial PV has not contributed to the* *development of this patient’s cancer. This specific test cannot exclude* *the possibility of an alternate PV in the tested gene or a PV in* *another cancer susceptibility gene in this patient. Further testing is* *available if required*.	

##### *6.4.5.2 Predictive testing*

For example, testing for a familial GENE PV in an individual without an HBOC-related cancer.

*6.4.5.2.1 Females*

*6.4.5.2.1.1 PV detected in high risk HBOC gene*

**Table:** 

See example wording 6.4.2.1.	

*6.4.5.2.1.2 PV detected in moderate risk HBOC gene or PV variant is of reduced penetrance*

**Table:** 

*This individual is at increased risk of developing GENE-related* *cancers. This variant <or gene> is associated with reduced <or* *moderate> penetrance <add reference> therefore patients should* *be managed appropriately, based on their personal and family* *history*. See also 6.4.1.1a for further wording.	

*6.4.5.2.1.3 Familial PV not detected*

**Table:** 

*This individual is* ***not*** *at increased risk of developing familial GENE-related* *cancer associated with this specific variant. Their residual risk* *for cancer is dependent on personal and familial history. Descendants* *are not at risk for the familial PV. Descendants are not at risk for the familial PV*.	

*6.4.5.2.2 Males*

Large differences in absolute cancer risk are reported for males (Tables S1 and S2), therefore it is considered acceptable not to give a numerical risk estimate and instead include a statement such as ‘specific risk figures for males should be discussed with clinical genetics’.

*6.4.5.2.2.1 BRCA1/PALB2 familial PV detected*

**Table:** 

*This individual is at increased risk of developing BRCA1/PALB2-* *related tumours; however, overall this risk is low (6.4.5.2.2)*. See 6.4.2.1 and 6.4.1.1a/b for further wording.	

*6.4.5.2.2.2 BRCA2 familial PV detected*

**Table:** 

*This individual is at increased risk of developing BRCA2-related tumours (6.4.5.2.2).* See 6.4.2.1 for further wording.	

*6.4.5.2.2.3 BRCA1/BRCA2/PALB2 familial PV not detected*

**Table:** 

*This individual is* ***not*** *at increased risk of developing familial GENE-related* *cancer associated with this specific variant. Descendants are* *not at risk for the familial PV*.	

### 6.5 Follow up

#### 6.5.1 Duty to re-report reclassified variants & performing report amendments

See [49]

See also Section 5.2: Variant review and reclassification

**Table:** 

49. *If the reclassification of a variant alters the clinical significance (i.e. from VUS to LP/LP to VUS), laboratories* ***must*** *assess if a reissue of a report to the referring consultant of the proband is required* [55]. 50. *Any reissued report* ***must*** *clearly state that it is an updated report which supersedes the previously issued report*.	

## 7.0 Discussion

Testing for HBOC has become increasingly complex. Gene associations attributed to moderate/low risk of disease are increasing and the addition of targeted therapy has introduced the need for somatic testing. Furthermore, variant classification for germline and somatic variants follow different guidelines. These HBOC testing guidelines have identified 50 recommendations and provide the diagnostic laboratory with current references for obtaining further information. Additionally, example report wording is suggested for ease of implementation.

### Supplementary information

Supplementary Material S2.1: Hereditary Breast Ovarian Cancer (HBOC) genes

Supplementary Material Table S1

Supplementary Material Table S2

Supplementary Material Table S3

References for Table S1, Table S2, Table S3 and Section S2.1

Supplementary Materials Table S4
