# Supplementary material for: EMQN best practice guidelines for genetic testing in hereditary breast and ovarian cancer
Source: Eur J Hum Genet. 2024 Mar 5;32(5):479–88. doi: 10.1038/s41431-023-01507-5 (PMC11061103; doi:10.1038/s41431-023-01507-5)
Supplement: Supplementary file 1 — Supplementary Material S2.1: Hereditary Breast Ovarian Cancer (HBOC) genes [file 41431_2023_1507_MOESM1_ESM.docx]

**S1.1 Hereditary Breast Ovarian Cancer (HBOC) genes**

**S1.1.1 High risk HBOC genes (>4-fold Relative Risk [RR])**

**S1.1.1.1 *BRCA1* and *BRCA2***

The cancer risk associated with *BRCA1/2* PVs is highest for female BC and OC. A definitive cancer risk association has also been demonstrated for *BRCA2* and male BC. Other *BRCA1/2*-associated cancers include pancreatic cancer and prostate cancer where in both cases, evidence of an association is strongest for *BRCA2* (Table S1).

The *BRCA1* gene comprises 23 exons, 22 of which are coding. At least 63 alternatively spliced transcripts have been identified (27). The *BRCA1* protein has three functional domains, one N-terminal RING domain and two C-terminal BRCT (*BRCA1*-C-terminal) domains. The protein has multiple cellular functions including DNA repair, cell cycle checkpoint control, transcriptional regulation, apoptosis and mRNA splicing. *BRCA1* maintains genome stability and acts as a tumour suppressor.

The *BRCA2* gene comprises 27 exons, 26 of which are coding. At least 24 alternatively spliced transcripts have been identified (28). The *BRCA2* protein has 8 BRC repeats, and a DNA binding domain that includes a helical domain and three oligonucleotide/oligosaccharide binding (OB) folds. It also binds with *PALB2* at the N-terminus. This protein is involved in the homologous recombination double-strand DNA repair pathway by binding *RAD51* and ssDNA and recruiting it to the site of DNA damage to enhance repair activity.

Germline PVs in *BRCA1/2* are inherited in an autosomal dominant (AD) manner. The *de novo* mutation rate for *BRCA1/2* is extremely low (<1%) (29). Biallelic inheritance is rare, but has been reported for both genes. Approximately 3-5% of cases of Fanconi anemia (FA-D1 group) are caused by biallelic PVs in the *BRCA2* gene, mostly in compound heterozygous form (30). In the case of *BRCA1*, biallelic inheritance in viable embryos is extremely rare, with only nine cases of compound heterozygosity in *BRCA1* reported to date (reviewed in (31)). The occurrence of a PV in both *BRCA1/2* in the same individual (transheterozygosity) has been reported to be present in approximately 0.3% of *BRCA1/2* cases in one large study (32). The most common occurrences involve founder PVs *BRCA1* c.68_69del p.(Glu23fs) and *BRCA2* c.5946del p.(Ser1982fs), comprising 33.3% of the 93 cases reported (32). There is currently no definitive evidence to support a specific phenotype associated with transheterozygosity.

Founder PVs have been identified in various populations. The five most common PVs, including founder PVs, have been reported to account for 33% of all PVs in *BRCA1* and 19% of all PVs in *BRCA2* (33).

Three well characterised founder PVs, namely c.68_69del p.(Glu23fs) and c.5266dup p.(Gln1756fs) (*BRCA1)* and c.5946del p.(Ser1982fs) (*BRCA2*) together account for more than 90% of all identified PVs in the Ashkenazi Jewish population. *BRCA1* c.68_69del p.(Glu23fs) and c.5266dup p.(Gln1756fs) are also commonly found in other populations. The *BRCA2* c.771_775del p.(Asn257fs) PV accounts for virtually all HBOC families in Iceland (33). Founder copy number variants (CNVs) have also been reported such as the *BRCA1* Alu-mediated tandem 6kb exon 13 duplication (legacy numbering). This pathogenic CNV is the 4th most common *BRCA1* PV in North America and comprises 9% of *BRCA1* PVs in the UK. It has also been found in other geographically diverse populations, such as Northern Sweden, Australia, Belgium, Canada, Italy, Norway (34).

Over 12,000 germline variants in *BRCA1/2* are recorded in the BRCA exchange database (<https://brcaexchange.org>). The majority of *BRCA1/2* PVs are protein truncating variants (PTVs) (in order of frequency: frameshift, nonsense, splice, and large deletion/duplication) and majority are subject to nonsense-mediated decay (33). *BRCA1/2* missense PVs are relatively infrequent, and are mostly located within highly conserved protein domains such as the RING (amino acid (aa) 2-101) or BRCT (aa 1650-1863) domains in BRCA1 and the DNA binding domain (aa 2481-3186) in BRCA2 ([https://enigmaconsortium.org](https://enigmaconsortium.org/)).

There is evidence to suggest that missense PVs in these BRCA1/2 critical domains may be associated with a reduced BC risk compared to PTVs, in particular for *BRCA1* for BC diagnosed >=50y (35,36).

Distinct from altering protein function, missense PVs can also affect mRNA splicing via different mechanisms e.g. *BRCA1* c.212G>A, p.(Arg71Lys) (creation of a new cryptic splice site); *BRCA2* c.8165C>G p.(Thr2722Arg) (disruption of exon splice enhancer (ESE) motifs), and *BRCA1* c.4484G>T p.(Arg1495Met) (affecting the last base of an exon).

A significant portion of PVs include CNVs spanning ≥1 exon. These are more common in *BRCA1* (10-15%) than *BRCA2* (2-4%) (14,33). Prevalence is higher in populations where there are founder CNVs. CNVs have been found to comprise approximately 20% of non-Jewish *BRCA1* PVs in the UK (37); and approximately 27% of all *BRCA1* PVs in the Dutch population (38).

Rare dominantly inherited 5’UTR variants causing *BRCA1* promoter hypermethylation and transcriptional silencing of the *BRCA1* gene have been identified (39,40). *BRCA1/2* germline variants which may affect gene regulation are increasingly being identified in promoter regions, and are likely to be identified in HBOC with the increasing use of whole genome sequencing (WGS). (39,41). These types of variants are not routinely screened for in current diagnostic testing panels; however, should future studies demonstrate increased prevalence, testing for these PVs would require consideration.

**S1.1.1.2. *PALB2***

After *BRCA1/2*, high risk PVs are most frequently identified in *PALB2* (Table S1).

*PALB2* (Partner and Localiser of *BRCA2*) interacts with *BRCA2* in nuclear foci, promotes its localisation and stability in nuclear structures, and enables the homologous recombination repair (HRR) and checkpoint functions of BRCA2 (42). It is considered a high risk predisposition gene for female BC, and there is supporting evidence for involvement in OC, male BC (Table S1) and pancreatic cancer (Table S2). The risk of OC is significantly lower than for *BRCA1/2* (Table S1). Data on cancer risk associated with *PALB2* to date is mostly based on PTVs (18). The contribution of missense variants is currently undetermined, with some evidence to suggest that the contribution may be minimal (36).

**S1.1.1.3 Other high risk HBOC genes**

*BRIP1, RAD51C,* and *RAD51D* are high risk OC predisposition genes involved in HRR. Recent studies (2,3,20) have confirmed a moderate risk of BC for *RAD51C* and *RAD51D PV* heterozygotes (Table S1)*;* as the risk is significantly modified by family history, an individualised risk evaluation can be performed using CanRisk. The same studies showed no significant association of *BRIP1* with BC (Table S2).

PVs in cancer syndrome genes *TP53*, *PTEN* and *STK11* contribute <1% to the overall prevalence of PVs identified in HBOC patient cohorts following MGP analysis (Table S1). However, as PVs in these genes are associated with a high absolute risk of BC; it is recommended that they are included on HBOC MGPs and that analysis is performed based on relevant criteria such as age of onset and clinical phenotype.

*CDH1,* a hereditary diffuse gastric cancer *(*HDGC) syndrome gene, may be considered a gene candidate for lobular BC (LBC) (Table S2); however, due to challenges in interpreting variants detected in the absence of a personal or family history of gastric cancer or LBC, inclusion in BC gene panel is not recommended (5). Clinical practice guidelines covering this topic have been published (43).

Lynch syndrome (LS), formerly known as hereditary non-polyposis colorectal cancer (HNPCC), is caused by PVs associated with multiple types of cancers, particularly colon, ovarian and endometrial/uterine. The contribution of *MLH1*, and in particular *MSH2* and *MSH6* to ovarian cancer is established (Table S1). PVs in these genes are the second highest cause of hereditary OC after *BRCA1/2*, accounting for 10–15% of hereditary OC cases (44) and are considered high risk OC predisposition genes. For *PMS2,* evidence is conflicting (Table S2). The contribution of these genes to hereditary BC is equivocal (Table S2).

**S1.1.2 Moderate risk HBOC genes (2-4 fold RR)**

After *BRCA1/2*, *CHEK2* PVs are most frequently detected in BC cases (Table S1).

PTV c.1100delC p.(Thr367fs) is the most common *CHEK2* PV identified in Western populations; however, it is rare or absent in other populations (45). Other commonly identified PVs include Polish founder variant *CHEK2* c.444+1G>A (46,47).

*CHEK2* missense variants may compromise approximately 30% of all variants identified (1). BC risk associated with *CHEK2* missense variants is generally lower, with estimated risks not reaching a clinically actionable threshold for the majority (2,3,36). As a result, reporting *CHEK2* missense variants is of limited clinical utility and not currently recommended (5). However, where sufficient data is available for specific variants e.g. c.349A>G (p.Arg117Gly) with OR 2.69 (1.46–4.94) or up to 3.40 (1.52–7.61) (if GWAS samples excluded) (36), reporting is recommended.

Risk of BC for PTVs in *ATM* is generally higher than for missense variants (48); however, there is evidence that for a subset of rare missense substitutions located within the FRAP-ATM-TRRAP (FAT) and phosphatidylinositol 3-kinase and 4-kinase (PIK) domains, the risk may be similar (36). Interestingly, this study did not show a significant association with BC risk for the widely reported variant c.7271T>G p.(Val2424Gly), for which an association higher than that for PTVs had been shown by other studies (49–52). This discordance may be due to subject ascertainment differences (36).

In the case of *BARD1*, estimates of female BC risk to date suggest a low to moderate risk. There is limited evidence in support for a role in OC (Table S2).

**S1.1.3. Candidate HBOC genes**

Whole exome sequencing (WES) and WGS studies are identifying new genetic loci for BC or OC susceptibility e.g. *MRE11*, *RAD50*, and *NBN*; however, diagnostic testing for these genes is not yet routine.
