# Supplementary material for: EMQN best practice guidelines for genetic testing in hereditary breast and ovarian cancer
Source: Eur J Hum Genet. 2024 Mar 5;32(5):479–88. doi: 10.1038/s41431-023-01507-5 (PMC11061103; doi:10.1038/s41431-023-01507-5)
Supplement: Supplementary file 2 — Supplementary Material Table S1 [file 41431_2023_1507_MOESM2_ESM.docx]

**Table S1: HBOC genes with an established associated cancer risk**

| **Symbol**   \| **Locus**  **OMIM gene**  **MANE Select Transcript**  **Mode of inheritance** \| \| --- \| | **PV case prevalence (%)** | **Relative cancer risk (95%CI) (p value)** | **Absolute cancer risk (95%CI)** |
| --- | --- | --- | --- | --- |
|  |  | **Female Breast cancer** |  |
| ***BRCA1***  17q21.31  113705  NM_007294.4  (AD/AR) | 2.21 (1)  1.00 (2)  0.85 (3) | OR 9.25 (8.20-10.30) (<0.0001) (1)  OR 10.57 (8.02-13.93) (<0.0001) (2)  OR 7.62 (5.33-11.27) (<0.001) (3)   - **NCCN (v1.2023) Evidence category for increased BC risk: Very Strong** - **Evidence category for increased BC risk** (4)**: Definitive** - **Agreed BC panel gene** (5) | >60% (6)  Primary: 72% by age 80y (65-79%), Contralateral: By 20y after diagnosis of primary: 40% (35-45%) (7)  ~55% by age 80y (2) |
|  |  | **Male Breast cancer** |  |
|  | 0.9 (8) | OR: 1.80 (0.30-6.80) (0.30) (8)  RR: 4.30 (1.09-16.96) (0.04) (9)  **NCCN (v1.2023) Evidence category for increased risk: Strong** | 0.2-1.2% (6)  1.2% (0.22-2.8%) to age 70y (10)  0.40% (0.10-1.50%) by age 80y (9)  0.50-1.50% (range from the bottom to the top PRS percentiles) (11) |
|  |  | **Ovarian cancer** |  |
|  | 8.61 (1)  3.97 (12) | OR 35.26 (29.60-42.00) (<0.0001) (1)  SRR 11.78 (10.42-13.28) (<0.0001) (12)   - **NCCN (v1.2023) Evidence category for increased OC risk: Very Strong** - **Evidence category for increased OC risk** (4)**: Definitive** - **Agreed OC panel gene** (5) | >39-58% (6)  44% by age 80y (36-55%) (7) |
|  |  | **Pancreatic cancer** |  |
|  | 0.60 (13)  0.80 (14) | OR 2.58 (1.54-4.05) (0.002) (13)  OR 2.07 (0.70-5.35) (0.14) (14)  RR 2.36 (1.51-3.68) (<0.001) (9)  **NCCN (v1.2023) Evidence category for increased risk: Strong** | ≤5% (6)  Males: 2.90% (1.90-4.50%) by age 80y (9)  Females: 2.30% (1.50-3.60%) by age 80y (9) |
|  |  | **Female Breast cancer** |  |
| ***BRCA2***  13q13.1  600185  NM_000059.4  (AD/AR) | 2.20 (1)  1.54 (2)  1.29 (3) | OR 5.67 (5.14-6.30) (<0.0001) (1)  OR 5.85 (4.85-7.06) (<0.0001) (2)  OR 5.23 (4.09-6.77) (0.001) (3)   - **NCCN (v1.2023) Evidence category for increased BC risk: Very Strong** - **Evidence category for increased BC risk** (4)**: Definitive** - **Agreed BC panel gene** (5) | >60% (6)  Primary: 69% by age 80y (61-77%)  Contralateral: By 20y after diagnosis of primary: 26% (20-33%) (7)  ~45% by age 80 (2)* |
|  |  | **Ovarian cancer** |  |
|  | 4.52 (1)  3.39 (12) | OR 11.91 (9.87-14.40) (<0.0001) (1)  SRR 7.97 (7.00-9.01) (<0.0001) (12)   - **NCCN (v1.2023) Evidence category for increased OC risk: Very Strong** - **Evidence category for increased OC risk** (4)**: Definitive** - **Agreed OC panel gene** (5) | 13-29% (6)  17% by age 80y (11-25%) (7) |
|  |  | **Male Breast cancer** |  |
|  | 8.1 (8) | OR 13.9 (8.50-22.50) (<0.0001) (8)  RR 44.03 (21.32-90.93) (<0.001) (9)  **NCCN (v1.2023) Evidence category for increased risk: Strong** | 1.80-7.10% (6)  7.1% (SE 5.2-8.6%) to age 70y; 8.4% (SE 6.2-10.6%) to age 80y (15)  6.8% (3.2-12%) by age 70y (10)  3.80% (1.9-7.7%) by age 80y (9)  5-14% (range from the bottom to the top PRS percentiles) (11) |
|  |  | **Pancreatic cancer** |  |
|  | 1.95 (13)  3.40 (14) | OR 6.20 (4.62-8.17) (<0.001) (13)  OR 7.13 (4.31-11.55) (5.52E-10) (14)  RR 3.34 (2.21-5.06) (<0.001) (9)  **NCCN (v1.2023) Evidence category for increased risk: Very Strong** | 5-10% (6)  Males: 3% (1.7-5.4%) by age 80y (9)  Females: 2.30% (1.3-4.2%) by age 80y (9) |
|  |  | **Prostate cancer** |  |
|  | 3.8 (16) | SIR 4.45 (2.99-6.61) relative to the population incidence (17)  RR 2.22 (1.63-3.03) (<0.001) (9)  *After adjusting for possible increased prostate-specific antigen screening effects:  SIR 2.34 (1.57-3.48) (17)  Previous RR estimates: 2-6 (17) & references therein | 19-61% (6)  27% by age 75y (95% CI 17-41%); 60%* by age 85y (95% CI 43-78%) (17)  *After adjusting for possible increased prostate-specific antigen screening effects, estimate = 41% (22-59%) (17)  26.90% (20.50-34.70%) by age 80y (9)  Previous retrospective studies: 15-34% by ages 65-80y (17) & references therein  19-61% (range from the bottom to the top PRS percentiles) (11) |
|  |  | **Female Breast cancer** |  |
| ***PALB2***  16p12.2  610355  NM_024675.4  (AD/AR) | 0.92 (1)  0.46 (3)  0.56 (2) | OR 4.87 (4·20-5·65) (<00001) (1)  RR: 7.18 (5.82-8.85) (<00001) (18)  OR: 3.83 (2.68-5.63) (<0.001) (3)  OR 5.02 (3.73-6.76) (<0.0001) (2)   - **NCCN (v1.2023) Evidence category for increased BC risk: Strong** - **Evidence category for increased BC risk** (4)**: Definitive** - **Agreed BC panel gene** (5) | 41-60% (6)  53% by age 80y (44-63%) (18)  ~40% by age 80y (2) |
|  |  | **Ovarian cancer** |  |
|  | 0.42 (1)  0.36 (12) | OR 2.13 (1·420-3·207) (0.0003) (1)  RR 2.91 (1.40-6.04) (4.1x10^-3^) (18)  SRR 3.08 (1.93-4.67) (1.2x10^-5^) (12)   - **NCCN (v1.2023) Evidence category for increased OC risk: Strong** - **Evidence category for increased OC risk** (4)**: Moderate** - **Not agreed OC gene** (5)*****   ****now proposed NHS GMS OC gene*** (<https://panelapp.genomicsengland.co.uk/>) | 3-5% (6)  5% by age 80y (2-10%) (18) |
|  |  | **Male Breast cancer** |  |
|  | 0.80 (8) | OR 6.60 (1.70-21.10) (0.013) (8)  RR: 7.34 (1.28-42.18) (2.6x12^-5^) (18)  **NCCN (v1.2023) Evidence category for increased risk : Strong** | 0.90% by age 70y (6)  1% by age 80y (0.20-5%) (18) |
|  |  | **Female Breast cancer** |  |
| ***ATM***  11q22.3  607585  NM_000051.4  (AD/AR) | 0.97 (1)  0.78 (3)  0.60 (2) | OR 2.42 (2·163-2·708) (<0.0001) (1)  OR 1.82 (1.46-2.27) (0.001) (3)  OR 2.10 (1.71-2.57) (<0.0001) (2)   - **NCCN (v1.2023) Evidence category for increased BC risk: Strong** - **Evidence category for increased BC risk** (4)**: Definitive** - **Agreed BC panel gene (truncating & c.7271T>G p.(Val2424Gly)** (5) | 20-40% (6)  ~22% by age 80y (2) |
|  |  | **Ovarian cancer** |  |
| ***BRIP1***  17q23.2  605882  NM_032043.3  (AD/AR) | 0.89 (19)  0.99 (12) | OR 4.94 (4.07-6.00) (<0.0001) (19)  SRR 4.99 (3.79-6.45) (<0.0001) (12)   - **NCCN (v1.2023) Evidence category for increased OC risk: Strong** - **Evidence category for increased OC risk** (4)**: Definitive** - **Agreed OC panel gene** (5) | 5-15% (6) |
|  |  | **Female Breast cancer** |  |
| ***CHEK2*****  22q12.1  604373  NM_007194.4  (AD) | 1.70 (1)  1.08 (3)  1.44 (2) | OR 2.47 (2.02-3.05) ((0.001) (3)  OR 2.54 (2.21-2.91) (<0.0001) (2)  (excludes: c.470T>C p.(Ile157Thr) and c.1283C>T p.(Ser428Phe))   - **NCCN (v1.2023) Evidence category for increased BC risk: Strong (based on frameshift PV))** - **Evidence category for increased BC risk** (4)**: Definitive** - **Agreed BC panel gene (truncating variants)** (5) | 20-40% (6)  ~ 24% by age 80y (2) |
|  |  | **Ovarian cancer (LS-Associated)** |  |
| ***MLH1***  3p22.2  120436  NM_000249.4  (AD) | 0.10 (1)  0.08 (12) | OR 1.44 (0.53-3.90) (0.68) (1)  SRR 2.20 (0.81-4.78) (0.12) (12)   - **NCCN (v1.2023) Evidence category for increased OC risk: Strong** - **Evidence category for increased OC risk** (4)**: Definitive** - **Agreed OC panel gene** (5) | 4-20% (6) |
|  |  | **Ovarian cancer (LS-Associated)** |  |
| ***MSH2***  2p21-p16.3  609309  NM_000251.3  (AD) | 0.24 (1)  0.38 (12) | OR 3.98 (1·818-8·695) (0.0007) (1)  SRR 13.91 (8.82-20.87) (<0.0001) (12)   - **NCCN (v1.2023) Evidence category for increased OC risk: Strong** - **Evidence category for increased OC risk** (4)**: Definitive** - **Agreed OC panel gene** (5) | 8-38% (6) |
|  |  | **Ovarian cancer (LS-Associated)** |  |
| ***MSH6***  2p16.3  600678  NM_000179.3  (AD) | 0.44 (1)  0.65 (12) | OR 4.08 (2·427-6·848) (<0.0001) (1)  SRR 5.04 (3.70-6.70) (<0.0001) (12)   - **NCCN (v1.2023) Evidence category for increased OC risk: Mixed** - **Evidence category for increased OC risk** (4)**: Definitive** - **Agreed OC panel gene** (5) | ≤1-13% (6) |
|  |  | **Female Breast cancer** |  |
| ***PTEN***  10q23.31  601728  NM_000314.8  (AD) | 0.07 (1)  0.03 (2)  0.02 (3) | OR 5.40 (3.15-9.23) (<0.0001) (1)  Insufficient data (3)  OR 2.25 (0.85-6.00) (0.1) (2)   - **NCCN (v1.2023) Evidence category for increased BC risk: Strong** - **Evidence category for increased BC risk** (4)**: Definitive** - **Agreed breast cancer panel gene** (5) | 40-60% (historical cohort data); >60% (projected estimates) (6) |
|  |  | **Ovarian cancer** |  |
| ***RAD51C***  17q22  602774  NM_058216.3  (AD/AR) | 0.63 (19)  0.79 (12) | RR 7.55 (5.60-10.19) (<0.0001) (20)  OR 5.59 (4.42-7.07) (<0.0001) (19)  SRR 5.12 (3.72-6.88) (<0.0001) (12)   - **NCCN (v1.2023) Evidence category for increased OC risk: Strong** - **Evidence category for increased OC risk** (4)**: Definitive** - **Agreed OC panel gene** (5) | 10-15% (6)  11% (6-21%) by age 80y (20) |
|  |  | **Female Breast cancer** |  |
|  | 0.15 (1)  0.11 (2)  0.13 (3) | RR 1.99 (1.39-2.85) (<0.0001) (20)  OR 1.93 (1.20-3.11) (0.07) (2)  OR 1.20 (0.75-1.93) (0.44) (3)   - **NCCN V.1.2023: Evidence category for increased BC risk: Strong** - **Agreed BC panel genes** (UKCGG meeting Oct 2021, Helen Hanson pers. comm.) | 20-40% (6)  21% (15-29%) by age 80y (20) |
|  |  | **Ovarian cancer** |  |
| ***RAD51D***  17q12  602954  NM_002878.4  (AD/AR) | 0.41 (19)  0.31 (12) | RR 7.60 (5.61-10.30) (<0.0001) (20)  OR 6.94 (4·028-13·140) (<0.0001) (19)  SRR 6.34 (5.10-9.44) (<0.0001) (12)   - **NCCN (v1.2023) Evidence category for increased OC risk: Strong** - **Evidence category for increased OC risk** (4)**: Definitive** - **Agreed OC panel gene** (5) | 10-20% (6)  13% (7-23%) by age 80y (20) |
|  |  | **Female Breast cancer** |  |
|  | 0.08 (1)  0.10 (2)  0.08 (3) | RR 1.83 (1.24-2.72) (0.002) (20)  OR 1.72 (0.88-3.51) (0.12) (3)  OR 1.80 (1.11-2.93) (0.018) (2)   - **NCCN V.1.2023: Evidence category for increased BC risk:Strong** - **Agreed BC panel genes** (UKCGG meeting Oct 2021, Helen Hanson pers. comm.) | 20-40% (6)  20% (14-28%) by age 80y (20) |
|  |  | **Female Breast cancer** |  |
| ***TP53***  17p13.1  191170  NM_000546.6  (AD) | 0.21 (1)  0.06 (3)  0.014 (2) | OR 4·36 (3.27-5.81) (<0.0001) (1)  OR NA (3)  OR 3.06 (0.63-14.91) (0.17) (2)   - **NCCN (v1.2023) Evidence category for increased BC risk: Strong** - **Evidence category for increased BC risk** (4)**: Definitive** - **Agreed BC panel gene** (5) | >60% (6) |

**Table S1 notes:**

1. Grey shading: High risk genes (>4-fold relative risk); No shading: Moderate risk genes (2-4-fold relative risk)
2. With the exception of *BRCA1, BRCA2 and PALB2*, genes are ordered alphabetically
3. Genes have been described using MANE select transcripts. MANE Plus Clinical Transcripts may be assigned in the future (21)
4. PV (pathogenic or likely pathogenic variant) prevalence refers to the total number of cases with a PV (LP/P) as a proportion (%) of the total number of cases tested
5. AD - autosomal dominant (with incomplete penetrance); AR - autosomal recessive
6. AD genes also associated with AR inheritance as part of the Fanconi anemia (FA) complementation group as follows (22)
   1. *BRCA2* FANCD1 (~3%)
   2. *BRIP1* – FANCJ (~2%);
   3. *PALB2* – FANCN
   4. *BRCA1* – FANCS
   5. *RAD51C* – FANCO
   6. *RAD51D* FANCR (individual case reports)
7. Biallelic PVs in *ATM* cause AR ataxia telangiectasia (A-T)
8. RR: Relative Risk, SIR: Standardised Incident Ratio, SRR: Standardised Rate Ratio, OR: Odds Ratio
9. See Table S3 Supplementary materials for source of material for Table S1
