# Supplementary material for: EMQN best practice guidelines for genetic testing in hereditary breast and ovarian cancer
Source: Eur J Hum Genet. 2024 Mar 5;32(5):479–88. doi: 10.1038/s41431-023-01507-5 (PMC11061103; doi:10.1038/s41431-023-01507-5)
Supplement: Supplementary file 3 — Supplementary Material Table S2 [file 41431_2023_1507_MOESM3_ESM.docx]

**Table S2: HBOC/candidate HBOC genes for which current cancer risk evidence is not definitive**

| **Symbol**   \| **Locus**  **OMIM gene**  **MANE Select Transcript**  **Mode of inheritance** \| \| --- \| | **PV case prevalence (%)** | **Relative cancer risk (95%CI) (p value)** | **Absolute cancer risk (95%CI)** |
| --- | --- | --- | --- | --- |
|  |  | **Prostate cancer** |  |
| ***BRCA1***  17q21.31  113705  NM_007294.4  (AD*/AR) | 0.80 (16) | SIR 2.35: (1.43-3.88) relative to the population incidence. At most, moderate risk: RR 2-4 <65y, 1-2 >65y*  *After adjusting for possible increased prostate-specific antigen screening effects:  SIR 1.24 (0.75-2.04); RR 0.82 (0.54-1.27) (0.38) (17)  Previous RR estimates: 0.3-4.0 (17), references therein | 7-26% (6)  21% by age 75y (95% Cl 3-34%); 29% by age 85y (95% Cl 17-45%)*  *After adjusting for possible increased prostate-specific antigen screening effects, estimate = 17% (8-26%) (17)  Previous retrospective studies: 3-9% by ages 65-80y (17), references therein  7-26% (range from the bottom to the top PRS percentiles) (11) |
|  |  | **Melanoma** |  |
|  | 0.80 (14) | OR 2.86 (significance not reached) (14)  RR 0.64 (0.14-2.95) (0.56) (9)  No statistically significant association demonstrated (23) | No definitive association |
|  |  | **Stomach** |  |
|  |  | RR 2.17 (1.25-3.77) (0.01) (9) | Males: 1.6% (0.7-4.0%) by age 80y (9)  Females: 0.7% (0.3-1.7%) by age 80y (9) |
|  |  | **Melanoma** |  |
| ***BRCA2***  13q13.1  600185  NM_000059.4  (AD*/AR) | 0.80 (14) | OR 2.18 (significance not reached) (14)  RR 0.93 (0.26-3.25) (0.91) (9)  Evidence for a statistically significant association is conflicting (23) | No definitive association |
|  |  | **Stomach** |  |
|  |  | RR 3.69 (2.40-5.67) (<0.001) (9) | Males: 3.5% (2.1-6.1%) by age 80y (9)  Females: 3.5% (1.9-6.4%) by age 80y (9) |
|  |  | **Pancreatic cancer** |  |
| ***PALB2***  16p12.2  610355  NM_024675.4  (AD*/AR) | 0.40 (13)  1.30 (14) | RR: 2.37 (1.24-4.50) (8.7x10^-3^) (18)  OR: 7.69 (3.88-14.44) (6.01E-07) (14)  **NCCN (v1.2023) Evidence category for increased risk: Limited** | 5-10% (6)  2-3% by age 80y (1-5%) (18) |
|  |  | **Ovarian cancer** |  |
| ***ATM***  11q22.3  607585  NM_000051.4  (AD/AR) | 0.70 (1)  0.87 (12) | OR 2.0 (1·330-2·939) (0.001) (1)  SRR 2.25 (1.69-2.94) (1.8x10^-7^) (12)   - **NCCN (v1.2023) Evidence category for increased OC risk : Strong** - **Evidence category for increased OC risk** (4)**: Limited** - **Not agreed OC panel gene** (5) | 2-3% (6) |
|  |  | **Female Breast cancer** |  |
| ***BARD1***  2q35  601593  NM_000465.4  (AD) | 0.21 (1)  0.27-0.83 (reviewed by (24))  0.13 (2)  0.15 (3) | OR 2·334 (1·834-2·969) (<0.0001) (1)  OR 2.00-5.35 (BC); 4.35-11.27 (TNBC) (reviewed by (24))  OR 2.09 (1.35-3.23) (0.001) (2)  OR 1.37 (0.87-2.16) (0.18) (3)   - **NCCN (v1.2023) Evidence category for increased BC risk: Strong** - **Evidence category for increased BC risk** (4)**: Definitive** - **Not agreed BC panel gene** (5) | 20-40% (6)  ~20% by age 80y (2) |
|  |  | **Ovarian cancer** |  |
|  | 0.14 (1)  0.14 (12) | OR 1.40 (0.685-2.90) (0.47) (1)  OR 0.14 (0.55-2.51) (0.59) (12)   - **NCCN V.1.2023: Evidence category for increased OC risk: None** - **Evidence category for increased OC risk** (4)**: Limited** - **Not an agreed OC panel gene** (5) | No established association (6) |
|  |  | **Female Breast cancer** |  |
| ***BRIP1***  17q23.2  605882  NM_032043.3  (AD*/AR) | <3% (1)  0.18 (2) | OR 1.11 (0.8-1.53) (0.54) (2)  OR 1·368 (1·153-1·624) (0.0004) (1)   - **NCCN V.1.2023: Evidence category for increased BC risk: Limited** - **Evidence category for increased BC risk** (4)**: Refuted** - **Not an agreed BC panel gene** (5) | Insufficient data to define (6) |
|  |  | **Female Breast cancer** |  |
| ***CDH1***  16q22.1  192090  NM_004360.5  (AD) | <0.2% (1)  0.02 (2)  0.05 (3) | OR 2.66 (1.678-4.20) (<0.0001) (1)  OR 2.50 (1.01-7.07) (0.06) (3)  OR 0.86 (0.37-1.98) (0.72) (2)   - **NCCN v. 1.2023: Evidence category for increased BC risk: Strong** - **Evidence category for increased BC risk** (4)**: Definitive** - **Not an agreed BC panel gene** (5) | 41-60% (6) |
|  |  | **Ovarian cancer** |  |
|  | 0.0 (1) | - **NCCN v. 1.2023: Evidence category for increased OC risk: None** - **Evidence category for increased OC risk** (4)**: No reported evidence** - **Not an agreed OC panel gene** (5) | No established association (6) |
|  |  | **Ovarian cancer** |  |
| ***CHEK2*****  22q12.1  604373  NM_007194.4  (AD) | 0.70 (1)  0.43 (12) | OR 0.43 (0·287-0.63) (0.84) (1)  SRR 0.98 (0.75-1.27) (0.87) (12)   - **NCCN V.2.2023: Evidence category for increased OC risk: None** - **Evidence category for increased OC risk** (4)**: Limited** - **Not an agreed OC panel gene** (5) | No established association (6) |
|  |  | **Female Breast cancer** |  |
| ***MLH1***  ***MSH2***  ***MSH6***  *(See Table S1)*  ***PMS2***  7p22  600259  NM_000535.7  (AD) | <0.30% (1)  (2) | MLH1: OR 0.68 (0.466-0.966) (0.050) (1)  MSH2: OR 1.67 (1.17-2.34) (0.0054) (1)  MSH6: OR 1.73 (1.37-2.2) (<0.0001) (1)  PMS2: OR 0.97 (0.787-1.2) (0.81) (1)  MLH1: OR 0.58 (0.19-1.77) (0.34) (2)  MSH2: OR 1.06 (0.47-2.36) (0.89) (2)  MSH6: OR 1.96 (1.15-3.33) (0.013) (2)  PMS2: OR 1.16 (0.73-1.85) (0.53) (2)   - **NCCN v 1.2023: Evidence category for increased BC risk: Limited** - **Evidence category for increased BC risk** (4)**: Disputed** - **Not agreed BC panel genes** (5) | <15% (6) |
|  |  | **Breast/Ovarian cancer** |  |
| ***MUTYH***  1p34.1  604933  NM_001048174.2  (AR) | BC: 0.042 (Biallelic) (1)  OC 0.0 (1)  BC 0.47 (2) | Insufficient data (1)  OR 1.00 (0.83-1.21) (0.99) (2)   - **Evidence category for increased BC risk** (4)**: No reported evidence (AD/AR)** - **Evidence category for increased OC risk** (4)**: Limited (AD/AR)** - **Not an agreed BC or OC panel gene** (5) | Insufficient evidence (6)  *The ClinGen Breast/Ovarian Gene Curation Expert Panel reviewed genetic and experimental evidence from published studies of MUTYH dominant (monoallelic) and recessive (biallelic) variants in breast and ovarian cancer. They found no reported evidence of MUTYH association with breast cancer and limited evidence for ovarian cancer susceptibility* |
|  |  | **Breast cancer** |  |
| ***NBN***  8q21.3  602667  NM_002485.5  (AD) | 0.083 (1)  0.184 (2) | OR 1.22 (0.98-1.53) (0.083) (1)  OR 0.90 (0.67-1.20) (0.48) (2)   - **Evidence category for increased BC risk** (4)**: Limited** - **Not an agreed BC panel gene** (5) | Insufficient evidence (6) |
|  |  | **Ovarian cancer** |  |
|  | 0.28 (1)  0.38 (12) | OR 2.12 (1.35-3.50) (0.002) (1)  SRR 2.03 (1.27-3.08) (0.004) (12)   - **Evidence category for increased OC risk** (4)**: Limited** - **Not an agreed OC panel gene** (5) | Insufficient Data |
|  |  | **Ovarian cancer (LS-Associated)** |  |
| ***PMS2***  7p22  600259  NM_000535.7  (AD) | 0.18 (1)  0.43 (12)) | 0.707 (0.29-1.7) (0.56) (1)  1.48 (0.81-2.48) (0.20) (12)   - **NCCN v 1.2023 Evidence category for increased OC risk: Limited** - **Evidence category for increased OC risk** (4)**: Disputed** - **Not agreed OC panel genes** (5) | 1.3-3% by age 80y (6) |
|  |  | **Ovarian Cancer** |  |
| ***PTEN***  10q23.31  601728  NM_000314.8  (AD) | 0.063 (1) | OR 5.47 (1.26-23.8) (0.08) (1)   - **NCCN v. 1.2023 Evidence category for increased OC risk: None** - **Evidence category for increased OC risk** (4)**: Disputed** - **Not agreed OC panel gene** (5) | No established association (6) |
|  |  | **Female Breast cancer** |  |
| ***STK11***  19p13.3  602216  NM_000455.5  (AD) | 0.01 (1)  0.012 (2) | OR 1.10 (0.324-3.80) (0.88) (1)  OR 1.60 (0.48-5.28) (2)   - **NCCN (v1.2023) Evidence category for increased BC risk: Strong**   **Evidence category for increased BC risk** (4)**: Definitive**   - **Agreed BC panel gene** (5) | 32-54% (6) |
|  |  | **Ovarian Cancer** |  |
|  | 0.0 (1) | Insufficient data for calculation of OR (1)   - **NCCN v. 1.2023 Evidence category for OC risk: None** - **Evidence category for increased OC risk** (4)**: No reported evidence** - **Not an agreed OC panel gene** (5) | No established association (6) |
|  |  | **Ovarian cancer** |  |
| ***TP53***  17p13.1  191170  NM_000546.6  (AD) | 0.3 (1) | OR 5.05 (2.41-10.58) (<0.0001) (1)   - **NCCN v. 1.2023 Evidence category for increased OC risk: None** - **Evidence category for increased OC risk** (4)**: Limited** - **Not agreed OC panel genes** (5) | No established association (6) |

**Table S2 notes:**

1. With the exception of *BRCA1, BRCA2 and PALB2*, genes are ordered alphabetically
2. Genes have been described using MANE select transcripts. MANE Plus Clinical Transcripts may be assigned in the future (21)
3. PV (pathogenic or likely pathogenic variant) prevalence refers to the total number of cases with a PV (LP/P) as a proportion (%) of the total number of cases tested
4. AD - autosomal dominant (with incomplete penetrance); AR - autosomal recessive
5. AD genes also associated with AR inheritance as part of the Fanconi anemia (FA) complementation group as follows (22)
   1. *BRCA2* FANCD1 (~3%)
   2. *BRIP1* – FANCJ (~2%);
   3. *PALB2* – FANCN
   4. *BRCA1* – FANCS
6. Biallelic PVs in *ATM* cause AR ataxia telangiectasia (A-T)
7. RR: Relative Risk, SIR: Standardised Incident Ratio, SRR: Standardised Rate Ratio, OR: Odds Ratio
8. See Table S3 Supplementary materials for source of material for Table S2
