# Supplementary material for: EMQN best practice guidelines for genetic testing in hereditary breast and ovarian cancer
Source: Eur J Hum Genet. 2024 Mar 5;32(5):479–88. doi: 10.1038/s41431-023-01507-5 (PMC11061103; doi:10.1038/s41431-023-01507-5)
Supplement: Supplementary file 4 — Supplementary Material Table S3 [file 41431_2023_1507_MOESM4_ESM.docx]

Table S3: Details of studies used for PV prevalence and associated cancer risks

| (1) | International study. Meta-analysis, 48 published MGP studies, 106529 Breast cancer patients (unselected and high risk cases), 3230 OC patients (unselected and high risk cases) tested for *BRCA1*/2; up to 7099 for *BRIP1/PALB2/BARD1*. Definition of PV: LOF (FS, N, +/1/+/-2 splice site), CNVs, missense + other intronic variants described as pathogenic in ClinVar (25) Associated risk calculated (OR): Comparison of PV (CNVs excluded) frequency in cases and 120,000 controls. Source of data for Table S1 and Table S2: Supplementary material, Table S4 (BC most and OC only) |
| --- | --- |
| (2) | International case control study. BCAC participants. Multigene panel (MGP) (34 genes). Population-based (unselected) 48826 cases, 50703 controls. Absolute risk in addition to associated risk (OR) calculated for PVs (protein-truncating variants). Source of data for Table S1 and Table S2: Table 1 (OR); Figure 3 (estimated absolute risk) |
| (3) | US CARRIERS Consortium case control study. MGP (28 genes). Population-based (unselected) 32247 cases, 32544 controls. Absolute risk (OR) calculated for PVs (LOF variants and variants classified as P/LP in ClinVar (25)). Source of data for Table S1 and Table S2: Table 2 (OR) |
| (4) | ClinGen Breast/Ovarian Gene Curation Expert Panel (GCEP) evaluation of 31 genes offered on HBOC MGP for evidence of association with breast and ovarian cancer. |
| (5) | UK Cancer Genetics Group guidelines for inclusion of genes on HBOC testing panels following input from clinicians and clinical scientists from NHS clinical genetics. |
| (6) | Referenced with permission from the NCCN Clinical Practice Guidelines in Oncology (NCCN Guidelines®) for Genetic/Familial High-Risk Assessment: Breast, Ovarian, and Pancreatic, v3.2023. © National Comprehensive Cancer Network, Inc. **2023.**  All rights reserved.  Accessed [April 25, 2023]**.**  To view the most recent and complete version of the guideline, go online to NCCN.org. NCCN makes no warranties of any kind whatsoever regarding their content, use or application and disclaims any responsibility for their application or use in any way. |
| (7) | Prospective study of breast and ovarian cancer risk in 6036 *BRCA1* and 3820 *BRCA2* female carriers of presumed pathogenic variants (VUS excluded) recruited through 3 consortia: IBCCS, BCFR, KConFab. Median follow-up: 5y. Cumulative breast and ovarian cancer risk to age 80y for *BRCA1*/2 presented |
| (8) | US retrospective Male BC study. Single clinical testing laboratory. MGP analysis + CNV analysis of 708 unselected MBC patients over 4 years. PV = P/LP as determined via ACMG guidelines (26). Associated risk calculated (OR): Comparison of PV frequency in cases and controls. Source of data for Table S1 and Table S2: Table 3, page 582 (Frequency of PVs - all MBC); Table 5, page 583 (OR). |
| (9) | Retrospective family-based study with self-reported cancer family history. Cancer risks associated with *BRCA1/2* PVs other than female breast and ovarian cancer. Definition of PV: <https://cimba.ccge.medschl.cam.ac.ul/eligibility/>. *BRCA1* families: 3184; *BRCA2* families: 2157. RR (SIR) calculated from observed cancer incidences with population incidence. Absolute risk estimates presented for ages 50, 60, 70 & 80y. RR data from Table 2, and absolute risk data from Table 4 presented |
| (10) | US retrospective multicentre male breast cancer study to determine the risk of MBC associated with *BRCA1/2*. Analysis of family history data collected on 1939 families. Most families were selected based on HBOC family history. 87 families reported MBC (23 MBC-*BRCA2*; 6 MBC-*BRCA1*). PV = FS + N. Relative and cumulative risk estimates obtained following statistical analysis of MBC carriers and non-carriers. Data source for Table S1 and Table S2: Table 2 |
| (11) | Case control study to determine whether prediction of BC or PC risk for male carriers of *BRCA1/2* can be stratified based on PRS. Male carriers recruited through the Consortium of Investigators of Modifiers of *BRCA1/2* (CIMBA). Definition of a PV: <https://cimba.ccge.medschl.cam.ac.ul/eligibility/>. Number of subjects: 1802: 277 BC, 212 PC, 1313 controls (no BC/PC). Male BC and PC absolute risks by age 80y presented for increasing percentiles of PRS distribution |
| (12) | US retrospective OC study. Single clinical testing lab. MGP (19 genes) + CNV analysis. 7768 unselected OC patients of European origin tested over 4 years. Significant cancer family history in 86.4%. PV = P/LP (FS, N, +/1/+/-2 splice site) and other variants reported as P/LP on ClinVar (25). Prevalence of PVs analysed and standardised risk ratio (SRR) calculated via case control analysis. Source of data for Table S1 and Table S2: Supplementary material, Table 2 |
| (13) | US retrospective pancreatic cancer study. Single clinical testing lab. MGP (21 genes). PV frequency in 3030 unselected pancreatic cancer patients tested over 6 years. Definition of PV: LOF (FS, N, +/1/+/-2 splice site) and missense + other intronic variants described as P/LP in ClinVar (25). Associated risk calculated (OR) via comparison of PV frequency in cases and controls. Data source for Table S1 and Table S2: Table 2 (overall PV frequency); Table 3 (OR) |
| (14) | US retrospective study. Single clinical testing lab. MGP (32 genes) + copy number variant (CNV) analysis. 165,000 BC/OC patients over approximately 4.75y. Most patients (90%) meeting NCCN V.1. 2018 criteria for HBOC testing. PV = P/LP as determined via ACMG guidelines (26). PV prevalence presented and associated PV cancer risks (OR) calculated via case control analysis. Data source for Table S1 and Table S2: Supplementary material Table S6 (OR), and Supplementary material Table S7, single primary cancer section (PV frequency). |
| (15) | UK prospective and retrospective analysis of 321 *BRCA2* families. Lifetime risk from prospective analysis via family ascertainment: 667 male first degree relatives with *BRCA2* PVs. Retrospective analysis: 508 male BRCA2 carriers/assumed carriers. Cumulative risk of BC estimated via Kaplan-Meier analysis (Figure 1). |
| (16) | US retrospective prostate cancer study. Single clinical testing lab. MGP (up to 67 genes) + CNV analysis. 1812 unselected prostate cancer cases tested over 5 years. Significant cancer family history in 92.4%. PV = P/LP as determined via ACMG guidelines (26). Source of data for Table S1: Figure 1, page 1519 (PV frequency) |
| (17) | UK multicentre prospective prostate cancer study (EMBRACE). *BRCA1*: 376 males (5.9y follow up), *BRCA2*: 447 males (5.3y follow-up). Observed prostate cancer incidence compared to that expected from population data (<https://www.ons.gov.uk>). to calculate standard incidence ratios calculated (SIR). Kaplan-Meier estimate to estimate absolute risks. PV = P as determined according to ENIGMA criteria (<https://enigmaconsortium.org>). Source of data for Table 1 = Table 2 (Overall data) |
| (18) | International retrospective study of 524 families with *PALB2* PVs. Analysis of RR associated with *PALB2* PVs and various cancers (relative to country-specific incidences) and absolute risk. PV = protein truncating only. Data source for Table S1 and Table S2: Table 1 (RR age-constant model) and Table 2 (Estimated Absolute Risk). |
| (19) | International study. Meta-analysis of 63 studies: 29,400 OC patients (unselected + high risk). Prevalence of PVs in *BRIP1*, *RAD51C, RAD51D* and estimation of OC risk (OR) by comparison of PVs in cases and 116,000 controls. Definition of PV: LOF (FS, N, +/-1/+/-2 splice site), CNVs, missense + other intronic variants described as pathogenic in ClinVar (25). Data source for Table S1: Table 1 |
| (20) | International retrospective study to ascertain estimates for relative and cumulative OC and BC cancer risk associated with PVs in *RAD51C* and *RAD51D*. Subjects: 125 families with *RAD51C* PV, 60 families with *RAD51D* PV. PV defined as FS/N, canonical splice site, large genomic deletions (variants in last exon excluded). RR and cumulative risk estimates calculated via complex segregation analysis, adjusting for ascertainment mode. |
| (23) | Literature review: Review of published studies from 1999 - 2012 investigating associations between *BRCA1/2* PV and melanoma/non-melanoma skin cancer risk. |
