# Supplementary material for: EMQN best practice guidelines for genetic testing in hereditary breast and ovarian cancer
Source: Eur J Hum Genet. 2024 Mar 5;32(5):479–88. doi: 10.1038/s41431-023-01507-5 (PMC11061103; doi:10.1038/s41431-023-01507-5)
Supplement: Supplementary file 5 — References for Table S1, Table S2, Table S3 and Section S2.1 [file 41431_2023_1507_MOESM5_ESM.docx]

References for Table S1, Table S2, Table S3 and Section S2.1

1. Suszynska M, Klonowska K, Jasinska AJ, Kozlowski P. Large-scale meta-analysis of mutations identified in panels of breast/ovarian cancer-related genes — Providing evidence of cancer predisposition genes. Gynecol Oncol [Internet]. 2019;153(2):452–62. Available from: https://doi.org/10.1016/j.ygyno.2019.01.027

2. Dorling L, Carvalho S, Allen J, González-Neira A, Luccarini C, Wahlström C, et al. Breast Cancer Risk Genes — Association Analysis in More than 113,000 Women. N Engl J Med. 2021;384(5):428–39.

3. Hu C, Hart SN, Gnanaolivu R, Huang H, Lee KY, Na J, et al. A Population-Based Study of Genes Previously Implicated in Breast Cancer. N Engl J Med. 2021;384(5):440–51.

4. Lee K, Seifert BA, Shimelis H, Ghosh R, Crowley SB, Carter NJ, et al. Clinical validity assessment of genes frequently tested on hereditary breast and ovarian cancer susceptibility sequencing panels. Genet Med [Internet]. 2019;21(7):1497–506. Available from: http://dx.doi.org/10.1038/s41436-018-0361-5

5. Taylor A, Brady AF, Frayling IM, Hanson H, Tischkowitz M, Turnbull C, et al. Consensus for genes to be included on cancer panel tests offered by UK genetics services: guidelines of the UK Cancer Genetics Group. J Med Genet. 2018;55(6):372–7.

6. (NCCN Clinical Practice Guidelines in Oncology (NCCN Guidelines®), Genetic/Familial High-Risk Assessment: Breast, Ovarian, and Pancreatic, v3.2023) (Daly et al. 2022). Genetic / Familial High-Risk Assessment : Breast , Ovarian , and Pancreatic. 2022;

7. Kuchenbaecker KB, Hopper JL, Barnes DR, Phillips KA, Mooij TM, Roos-Blom MJ, et al. Risks of breast, ovarian, and contralateral breast cancer for BRCA1 and BRCA2 mutation carriers. JAMA - J Am Med Assoc. 2017;317(23):2402–16.

8. Pritzlaff M, Summerour P, McFarland R, Li S, Reineke P, Dolinsky JS, et al. Male breast cancer in a multi-gene panel testing cohort: insights and unexpected results. Breast Cancer Res Treat. 2017;161(3):575–86.

9. Li S, Silvestri V, Leslie G, Rebbeck TR, Neuhausen SL, Hopper JL, et al. Cancer Risks Associated With BRCA1 and BRCA2 Pathogenic Variants . J Clin Oncol. 2022;14–7.

10. Tai YC, Domchek S, Parmigiani G, Chen S. Breast cancer risk among male BRCA1 and BRCA2 mutation carriers. J Natl Cancer Inst. 2007;99(23):1811–4.

11. Lecarpentier J, Kuchenbaecker KB, Barrowdale D, Dennis J, McGuffog L, Leslie G, et al. Prediction of breast and prostate cancer risks in male BRCA1 and BRCA2 mutation carriers using polygenic risk scores. J Clin Oncol. 2017;35(20):2240–50.

12. Lilyquist J, LaDuca H, Polley E, Davis BT, Shimelis H, Hu C, et al. Frequency of mutations in a large series of clinically ascertained ovarian cancer cases tested on multi-gene panels compared to reference controls. Gynecol Oncol. 2017;147(2):375–80.

13. Hu C, Hart SN, Polley EC, Gnanaolivu R, Shimelis H, Lee KY, et al. Association between inherited germline mutations in cancer predisposition genes and risk of pancreatic cancer. In: JAMA - Journal of the American Medical Association. 2018.

14. LaDuca H, Polley EC, Yussuf A, Hoang L, Gutierrez S, Hart SN, et al. A clinical guide to hereditary cancer panel testing: evaluation of gene-specific cancer associations and sensitivity of genetic testing criteria in a cohort of 165,000 high-risk patients. Genet Med. 2020;

15. Evans DGR, Susnerwala I, Dawson J, Woodward E, Maher ER, Lalloo F. Risk of breast cancer in male BRCA2 carriers. J Med Genet. 2010;47(10):710–1.

16. Pritzlaff M, Tian Y, Reineke P, Stuenkel AJ, Allen K, Gutierrez S, et al. Diagnosing hereditary cancer predisposition in men with prostate cancer. Genet Med [Internet]. 2020;22(9):1517–23. Available from: http://dx.doi.org/10.1038/s41436-020-0830-5

17. Nyberg T, Frost D, Barrowdale D, Evans DG, Bancroft E, Adlard J, et al. Prostate Cancer Risks for Male BRCA1[Formula presented] and BRCA2 Mutation Carriers: A Prospective Cohort Study. Eur Urol. 2020;77(1):24–35.

18. Yang X, Leslie G, Doroszuk A, Schneider S, Allen J, Decker B, et al. Cancer risks associated with germline PALB2 pathogenic variants: An international study of 524 families. J Clin Oncol. 2020;38(7):674–85.

19. Suszynska M, Ratajska M, Kozlowski P. BRIP1, RAD51C, and RAD51D mutations are associated with high susceptibility to ovarian cancer: Mutation prevalence and precise risk estimates based on a pooled analysis of ~30,000 cases. J Ovarian Res. 2020;13(1):1–11.

20. Yang X, Song H, Leslie G, Engel C, Hahnen E, Auber B, et al. Ovarian and Breast Cancer Risks Associated with Pathogenic Variants in RAD51C and RAD51D. J Natl Cancer Inst. 2020;112(12):1242–50.

21. Morales J, Pujar S, Loveland JE, Astashyn A, Bennett R, Berry A, et al. A joint NCBI and EMBL-EBI transcript set for clinical genomics and research. Nature [Internet]. 2022;604(7905):1–6. Available from: http://dx.doi.org/10.1038/s41586-022-04558-8

22. Mehta PA, Tolar J. Fanconi Anemia Summary Genetic counseling. Gene Rev [Internet]. 2018;0(0):1–42. Available from: https://www.ncbi.nlm.nih.gov/books/NBK1401/pdf/Bookshelf_NBK1401.pdf

23. Gumaste P V., Penn LA, Cymerman RM, Kirchhoff T, Polsky D, McLellan B. Skin cancer risk in BRCA1/2 mutation carriers. Br J Dermatol. 2015;172(6):1498–506.

24. Alenezi WM, Fierheller CT, Recio N, Tonin PN. Literature review of BARD1 as a cancer predisposing gene with a focus on breast and ovarian cancers. Genes (Basel). 2020;11(8):1–24.

25. Landrum MJ, Lee JM, Benson M, Brown GR, Chao C, Chitipiralla S, et al. ClinVar: Improving access to variant interpretations and supporting evidence. Nucleic Acids Res. 2018;46(D1):D1062–7.

26. Richards S, Aziz N, Bale S, Bick D, Das S, Gastier-Foster J, et al. Standards and guidelines for the interpretation of sequence variants: a joint consensus recommendation of the American College of Medical Genetics and Genomics and the Association for Molecular Pathology. 2015; Available from: www.lrg-sequence.org

27. Colombo M, Blok MJ, Whiley P, Santamariñ M, Gutié Rrez-Enríquez S, Romero A, et al. Comprehensive annotation of splice junctions supports pervasive alternative splicing at the BRCA1 locus: a report from the ENIGMA consortium. Hum Mol Genet [Internet]. 2014;23(14):3666–80. Available from: https://academic.oup.com/hmg/article/23/14/3666/556399

28. Fackenthal JD, Yoshimatsu T, Zhang B, de Garibay GR, Colombo M, Vecchi G De, et al. Naturally occurring BRCA2 alternative mRNA splicing events in clinically relevant samples. J Med Genet. 2016;

29. Golmard L, Delnatte C, Laugé A, Moncoutier V, Lefol C, Abidallah K, et al. Breast and ovarian cancer predisposition due to de novo BRCA1 and BRCA2 mutations. Oncogene. 2016;

30. Meyer S, Tischkowitz M, Chandler K, Gillespie A, Birch JM, Evans DG. Fanconi anaemia, BRCA2 mutations and childhood cancer: A developmental perspective from clinical and epidemiological observations with implications for genetic counselling. J Med Genet. 2014;51(2):71–5.

31. Chirita-Emandi A, Andreescu N, Popa C, Mihailescu A, Riza AL, Plesea R, et al. Biallelic variants in BRCA1 gene cause a recognisable phenotype within chromosomal instability syndromes reframed as BRCA1 deficiency. J Med Genet. 2021;58(9):648–52.

32. Rebbeck TR, Friebel TM, Mitra N, Wan F, Chen S, Andrulis IL, et al. Inheritance of deleterious mutations at both BRCA1 and BRCA2 in an international sample of 32,295 women. Breast Cancer Res [Internet]. 2016;18(1). Available from: http://dx.doi.org/10.1186/s13058-016-0768-3

33. Rebbeck TR, Friebel TM, Friedman E, Hamann U, Huo D, Kwong A, et al. Mutational spectrum in a worldwide study of 29,700 families with BRCA1 or BRCA2 mutations. Hum Mutat. 2018;

34. Edwinsdotter Ardnor C, Rosén A, Ljuslinder I, Melin B. The BRCA1 exon 13 duplication: clinical characteristics of 22 families in Northern Sweden. Fam Cancer [Internet]. 2019;18(1):37–42. Available from: http://dx.doi.org/10.1007/s10689-018-0098-y

35. Li H, Engel C, de la Hoya M, Peterlongo P, Yannoukakos D, Livraghi L, et al. Risks of breast and ovarian cancer for women harboring pathogenic missense variants in BRCA1 and BRCA2 compared with those harboring protein truncating variants. Genet Med. 2022;24(1):119–29.

36. Dorling L, Carvalho S, Allen J, Parsons MT, Fortuno C, González-Neira A, et al. Breast cancer risks associated with missense variants in breast cancer susceptibility genes. Genome Med [Internet]. 2022;14(1):1–17. Available from: https://doi.org/10.1186/s13073-022-01052-8

37. Smith MJ, Urquhart JE, Harkness EF, Miles EK, Bowers NL, Byers HJ, et al. The Contribution of Whole Gene Deletions and Large Rearrangements to the Mutation Spectrum in Inherited Tumor Predisposing Syndromes. Hum Mutat. 2016;37(3):250–6.

38. Hogervorst FBL, Nederlof PM, Gille JJP, McElgunn CJ, Grippeling M, Pruntel R, et al. Large genomic deletions and duplications in the BRCA1 gene identified by a novel quantitative method. Cancer Res. 2003;63(7):1449–53.

39. Burke LJ, Sevcik J, Gambino G, Tudini E, Mucaki EJ, Shirley BC, et al. BRCA1 and BRCA2 5′ noncoding region variants identified in breast cancer patients alter promoter activity and protein binding. Hum Mutat. 2018;39(12):2025–39.

40. Evans DGR, van Veen EM, Byers HJ, Wallace AJ, Ellingford JM, Beaman G, et al. A Dominantly Inherited 5′ UTR Variant Causing Methylation-Associated Silencing of BRCA1 as a Cause of Breast and Ovarian Cancer. Am J Hum Genet [Internet]. 2018;103(2):213–20. Available from: https://doi.org/10.1016/j.ajhg.2018.07.002

41. dos Santos ES, Lallemand F, Burke L, Stoppa-Lyonnet D, Brown M, Caputo SM, et al. Non-coding variants in BRCA1 and BRCA2 genes: Potential impact on breast and ovarian cancer predisposition. Cancers (Basel). 2018;10(11):1–21.

42. Nepomuceno TC, De Gregoriis G, de Oliveira FMB, Suarez-Kurtz G, Monteiro AN, Carvalho MA. The role of PALB2 in the DNA damage response and cancer predisposition. Int J Mol Sci. 2017;18(9):1–20.

43. Blair VR, McLeod M, Carneiro F, Coit DG, D’Addario JL, van Dieren JM, et al. Europe PMC Funders Group Hereditary Diffuse Gastric Cancer : Updated Clinical Practice Guidelines. Lancet Oncol [Internet]. 2020;21(8):1–32. Available from: /pmc/articles/PMC7116190/%0A/pmc/articles/PMC7116190/?report=abstract%0Ahttps://www.ncbi.nlm.nih.gov/pmc/articles/PMC7116190/

44. Toss A, Tomasello C, Razzaboni E, Contu G, Grandi G, Cagnacci A, et al. Hereditary ovarian cancer: Not only BRCA 1 and 2 Genes. Biomed Res Int. 2015;2015(Figure 1).

45. Apostolou P, Papasotiriou I. Current perspectives on CHEK2 mutations in breast cancer. Breast Cancer Targets Ther. 2017;9:331–5.

46. Cybulski C, Wokołorczyrk D, Huzarski T, Byrski T, Gronwald J, Górski B, et al. A large germline deletion in the Chek2 kinase gene is associated with an increased risk of prostate cancer. J Med Genet. 2006;43(11):863–6.

47. Walsh T, Casadei S, Coats KH, Swisher E, Stray SM, Higgins J, et al. Spectrum of mutations in BRCA1, BRCA2, CHEK2, and TP53 in families at high risk of breast cancer. J Am Med Assoc. 2006;295(12):1379–88.

48. Girard E, Eon-Marchais S, Olaso R, Renault AL, Damiola F, Dondon MG, et al. Familial breast cancer and DNA repair genes: Insights into known and novel susceptibility genes from the GENESIS study, and implications for multigene panel testing. Int J Cancer. 2019;144(8):1962–74.

49. Gatti RA, Tward A, Concannon P. Cancer risk in ATM heterozygotes: A model of phenotypic and mechanistic differences between missense and truncating mutations. Mol Genet Metab. 1999;

50. Goldgar DE, Healey S, Dowty JG, Silva L Da, Chen X, Spurdle AB, et al. Rare variants in the ATM gene and risk of breast cancer. Breast Cancer Res [Internet]. 2011;13(4):R73. Available from: http://breast-cancer-research.com/content/13/4/R73

51. Tavtigian S V., Oefner PJ, Babikyan D, Hartmann A, Healey S, Le Calvez-Kelm F, et al. Rare, Evolutionarily Unlikely Missense Substitutions in ATM Confer Increased Risk of Breast Cancer. Am J Hum Genet. 2009;85(4):427–46.

52. van Os NJH, Roeleveld N, Weemaes CMR, Jongmans MCJ, Janssens GO, Taylor AMR, et al. Health risks for ataxia-telangiectasia mutated heterozygotes: a systematic review, meta-analysis and evidence-based guideline. Clin Genet. 2016;90(2):105–17.
