# Supplementary material for: EMQN best practice guidelines for genetic testing in hereditary breast and ovarian cancer
Source: Eur J Hum Genet. 2024 Mar 5;32(5):479–88. doi: 10.1038/s41431-023-01507-5 (PMC11061103; doi:10.1038/s41431-023-01507-5)
Supplement: Supplementary file 6 — Supplementary Materials Table S4 [file 41431_2023_1507_MOESM6_ESM.docx]

**Table S4: HBOC Genetic Testing Best Practice Recommendations**

| **Rec#** | **Main document Section** | **Recommendations** |
| --- | --- | --- |
|  |  | **HBOC REFERRAL PATHWAYS** |
| 1 | 3.1 | Appropriate genetic counselling must be an integral part of the process for individuals undergoing predictive testing. |
| 2 | 3.1 | Clinical genetics involvement should be recommended for the small number of patients who have secondary or incidental findings e.g. copy number loss or gain identified by array analysis, or PVs identified through whole genome/exome/direct-to-consumer tests. |
| 3 | 3.2 | In addition to ovarian tumour, breast tumour and other HBOC-related tumour testing should also be utilised as PARPi therapies are licensed and available. |
|  |  | **GENETIC TESTING** |
| 4 | 4.0 | Laboratories must ensure that the performance of analytical methods meets the required standard for diagnostic testing through initial and ongoing internal validation/verification and appropriate EQA participation. |
| 5 | 4.1 | As a minimum, laboratories must ensure that a HBOC diagnostic testing service (internal or via an external testing laboratory referral) includes analysis of high risk genes *BRCA1/2* and *PALB2*.  *Linked reporting recommendation (34)* |
| 6 | 4.1 | Laboratories must remain vigilant with current scientific literature and guideline updates to ensure MGPs remain current. |
| 7 | 4.1 | Laboratories should ensure that a HBOC diagnostic testing service (internal or via an external testing laboratory referral) provides analysis of intronic regions known to contain recurrent PVs.  *Linked reporting recommendation (34)* |
| 8 | 4.1 | Reportable variants detected in genes with associated pseudogenes (for example, *CHEK2* and *PMS2*) must be confirmed for specificity prior to reporting. |
| 9 | 4.1 | Laboratories should have policies in place for determining which genes are analysed for CNVs |
| 10 | 4.1 | As a minimum, laboratories must ensure that a HBOC diagnostic testing service (internal or via an external testing laboratory referral) provides CNV analysis for *BRCA1/2.*  *Linked reporting recommendation (34)* |
| 11 | 4.1.1.1 | When performing tumour testing, laboratories must establish the assay LoD and analytical sensitivity for all relevant variant types through appropriate validation.  *Linked reporting recommendation (32)* |
| 12 | 4.1.1.1 | When performing tumour testing, the percentage neoplastic cell content assessment must be integral to this analysis.  *Linked reporting recommendation (44)* |
| 13 | 4.1.1.1 | The neoplastic cell content percentage should be at least twice the validated limit of detection of the assay (LoD).  *Linked reporting recommendation (44)* |
| 14 | 4.1.1.1 | When performing PARPi-directed testing, paired germline and tumour analysis should ideally be performed, with germline testing involving full gene panel analysis as appropriate for the tumour type, in addition to analysis of larger indels and CNVs.  *Linked reporting recommendation 42, 43, 45, 46, 47* |
| 15 | 4.1.1.1 | When performing PARPi-directed testing using a tiered analysis protocol starting with somatic tissue; at a minimum, targeted germline follow-up testing should be performed to confirm any detected somatic PVs with VAF>30% (SNVs) or >20% (small indels), and to detect larger indels and CNVs which may have a lower LoD |
| 16 | 4.2 | Confirmation of the correct gene region to be targeted must be integral to the predictive testing process. |
| 17 | 4.2 | A familial positive control where available (ideally first degree relative) should be included with each assay to minimise the risk of a false negative result.  *Linked reporting recommendation (39)* |
| 18 | 4.2 | Primer/probe sequences must be checked regularly against recent large population studies (e.g. gnomAD) to ensure there are no reported SNVs which could potentially cause non-amplification of one allele. |
| 19 | 4.2 | To minimise the risk of incidental findings, analysis for a specific variant should be limited to a defined region of the gene containing the variant, including when NGS technology is used.  *Linked reporting recommendation (38)* |
|  |  | **VARIANT INTERPRETATION** |
| 20 | 5.1 | Germline variant classification must be performed according to ACMG/AMP guidelines (or national/local approved guidelines) with use of gene-specific and expert guidelines where available e.g. ClinGen VCEP, CanVIG or ENIGMA.  *Linked reporting recommendation (28)* |
| 21 | 5.1 | Variants identified during somatic testing should be classified using both ACMG/AMP germline guidance and somatic tiering. |
| 22 | 5.1 | Variants identified should be submitted to a database such as ClinVar to aid review and classification. |
| 23 | 5.2 | Laboratories must consider a variant review after a laboratory-defined period of variant classification has lapsed (e.g. > 12 months) and:   1. Re-identification of the variant in the laboratory OR 2. Following the release of new information from another laboratory or from the scientific literature/public database (ClinVar/Decipher) OR 3. Following new phenotypic information on the proband or family member OR 4. Following a request from the referring consultant e.g. prior to cascade testing, risk-reducing surgery etc.   *Linked reporting recommendation (49, 50)* |
| 24 | 5.2 | Where reclassification could change clinical management, and particularly if the evidence is not publicly available, notification should be provided to relevant other diagnostic laboratories and appropriate healthcare professionals.  *Linked reporting recommendation (49, 50)* |
| 25 | 5.3 | For *TP53* variants, the presence of CHIP/mosaicism must be considered as appropriate depending on familial transmission, VAF and phenotype.  *Linked reporting recommendation (35)* |
| 26 | 5.3 | Laboratories should perform analysis to exclude/confirm CHIP.  *Linked reporting recommendation (35)* |
| 27 | 5.3 | Caution should be exercised when reporting missense variants in genes *CHEK2,* *ATM* and *PALB2,* where disease has been predominantly associated with PTVs. |
|  |  | **REPORTING** |
| 28 | 6.1 | Guidelines used for variant interpretation must be clearly referenced in clinical reports. |
| 29 | 6.1 | Variant classification evidence must be available to service users, preferably as part of the report, or minimally upon request. |
| 30 | 6.2 | When a reportable germline variant is identified, laboratory reports must recommend appropriate clinical management and genetic counselling |
| 31 | 6.2 | Where an elevated residual risk remains after testing, and depending on local reporting policy: Reports should advise that clinical management should be dependent on personal and family history |
| 32 | 6.3 | Technical information including NGS/MLPA kit details and version number, sequencing chemistry, bioinformatics pipeline, LoD, analytical sensitivity must be available via the report. |
| 33 | 6.3 | Each variant reported must be described using HGVS nomenclature, including the clinically appropriate transcript (e.g. MANE select and/or MANE Plus Clinical) and version number, zygosity (germline variants), and include details of the predicted effect on the protein where appropriate. |
| 34 | 6.3.1 | The report must state the test scope and assay limitations, and refer the patient elsewhere for further testing/analysis of additional genes implicated in HBOC as appropriate, should the analytical and/or clinical sensitivity fail to reach the required laboratory-determined threshold/standard. |
| 35 | 6.3.1 | For *TP53* variants fitting the criteria for consideration of CHIP/mosaicism (see section 5.3), reports must clearly state the risk of CHIP if further work is not performed, or CHIP has been experimentally excluded/confirmed. |
| 36 | 6.3.1 | Variant classes not reported must be clearly stated on the report. |
| 37 | 6.3.1 | If a PV is identified in a patient where consanguinity has been noted, the report should mention the risk of AR disease e.g. FA and offer to test any consanguineous partner. |
| 38 | 6.3.2 | When performing specific variant testing (for example, predictive testing), the extent of the gene region analysed must be clear in the report. |
| 39 | 6.3.2 | When performing specific variant testing (for example, predictive testing) without a familial control, this limitation must be stated in PV-absent reports. |
| 40 | 6.4. | See specific HBOC reporting recommendations: 6.4.1, 6.4.2, 6.4.3, 6.4.4, 6.4.5 |
| 41 | 6.4.4.1 | When performing PARPi-directed somatic/germline testing, referral for appropriate clinical management and genetic counselling must be recommended for all reportable variants identified during germline testing. |
| 42 | 6.4.4.1 | When performing PARPi-directed testing, the report must clearly state whether variants identified were found in the somatic or germline test. |
| 43 | 6.4.4.1 | When solely performing PARPi-directed somatic testing, reports must recommend germline testing of the detected reportable variant, if VAF is in the laboratory established germline range |
| 44 | 6.4.4.1 | When performing PARPi-directed somatic testing, reports must clearly indicate the percentage of neoplastic cell content, and whether it is below the laboratory-determined acceptable threshold. |
| 45 | 6.4.4.1 | When performing PARPi-directed testing, reports must clearly state whether analysis for CNVs/larger indels has been performed. |
| 46 | 6.4.4.1 | Germline CNV/larger indel analysis for *BRCA1/2* should be recommended if this is not completed during PARPi-directed somatic testing (unless HRD negative) and the limitations of somatic-only testing should be clearly stated. |
| 47 | 6.4.4.1 | Somatic testing should be recommended if PARPi-directed germline testing has not identified a reportable variant, and the limitations of germline-only testing clearly stated. |
| 48 | 67.4.4.1 | As applicable, reports should refer to PARPi therapy rather than to brand names |
| 49 | 6.5.1 | Following variant review, if the reclassification of a variant alters the clinical significance (i.e. from VUS to LP/LP to VUS), laboratories must assess if a reissue of a report to the referring consultant of the proband is required. |
| 50 | 6.5.1 | Any reissued reports must clearly state that the new report supersedes the former report. |

**Table S4 notes**Guidelines are provided in the form of recommendations where ‘must’ is assigned to advocate that the recommendation is essential; and ‘should’ is assigned to advocate that the recommendation is highly advised but may not be universally applicable.
